# The long non-coding RNA CYTOR drives colorectal cancer progression by interacting with NCL and Sam68

**DOI:** 10.1186/s12943-018-0860-7

**Published:** 2018-07-31

**Authors:** Xue Wang, Hongfei Yu, Wenjie Sun, Jianlu Kong, Lei Zhang, Jinlong Tang, Jingyu Wang, Enping Xu, Maode Lai, Honghe Zhang

**Affiliations:** 1 Department of Pathology, Key Laboratory of Disease Proteomics of Zhejiang Province, Zhejiang University School of Medicine, Hangzhou, 310058 China; 2 Department of Pharmacology, China Pharmaceutical University, Nanjing, 210009 China

**Keywords:** Colorectal cancer, CYTOR, NCL, Sam68

## Abstract

**Background:**

Long non-coding RNAs (lncRNAs) function as key molecules in cancer progression. The lncRNA CYTOR plays oncogenic roles in multiple types of cancer, yet the detailed molecular mechanisms of those roles remain unknown. The aim of this study was to investigate the clinical significance, biological function and interacting partners of CYTOR in colorectal cancer (CRC).

**Methods:**

A systematic and comprehensive analysis of CYTOR expression was performed in 138 CRC samples and in the TCGA and GEO databases. Biological function was investigated through knockdown and overexpression of CYTOR in vitro and in vivo. In addition, its protein binding partner was identified and validated using ChIRP-MS and RNA immunoprecipitation assays. Their key interaction sites on CYTOR were verified by CRISPR/Cas9 and a series of mutant constructs. Furthermore, the downstream targets of CYTOR were confirmed via immunoblotting and luciferase reporter assays.

**Results:**

CYTOR was significantly up-regulated in CRC samples and associated with poor prognosis, promoting proliferation and metastasis in vitro and in vivo. NCL and Sam68 could recognize their specific motifs and directly bind to EXON1 of CYTOR. Moreover, EXON1 was the key functional site mediating the interaction of CYTOR with NCL and Sam68. NCL and Sam68 functioned as oncogenes to promote CRC progression. Furthermore, we confirmed that the heterotrimeric complex of CYTOR, NCL and Sam68 activated the NF-κB pathway and EMT to contribute to CRC progression.

**Conclusion:**

CYTOR plays important roles in CRC progression by interacting with NCL and Sam68 and may serve as a prognostic biomarker and/or an effective target for CRC therapies.

**Electronic supplementary material:**

The online version of this article (10.1186/s12943-018-0860-7) contains supplementary material, which is available to authorized users.

## Background

It is now widely accepted that cancer progression results from the gradual accumulation of genetic mutations and epigenetic alterations that successively increase cell proliferation, motility and stemness [1, 2]. Long non-coding RNAs (lncRNAs), since their recent discovery, have gained widespread attention as a new player of epigenetic regulation in various cellular and biological processes including gene regulation and chromatin dynamics; and the aberrant expression and mutations of lncRNAs are closely linked to tumorigenesis, metastasis, and tumor stage [3].

Colorectal cancer (CRC) is a frequently diagnosed malignancy and the 3rd leading cause of cancer-related mortality in the western world. The progression of CRC is fast, and untreated tumors rapidly disseminate and form metastatic foci. Therefore, further understanding the mechanisms that drive the disease and identifying diagnostic and therapeutic targets are priorities for improving CRC patients’ outcome [4]. To date, several important lncRNAs such as UPAT [5], CCAT1 [6], CCAL [7], LINC01133 [8], LET [9] and HOTAIR [10] have been shown to contribute to CRC development and could be used as new candidates for diagnostics and therapy. Previously, we found through laser microdissection capture in CRC tissues that a novel lncRNA, CYTOR (also known as LINC00152), was up-regulated in tumor budding cells [11]. Recently, the aberrant expression of CYTOR has been reported in some types of cancers including gastric cancer [12], hepatocellular carcinoma [13], colon cancer [14], gallbladder cancer [15] and renal cell carcinoma [16], in which it may act as an oncogene. However, the detailed mechanism of action of CYTOR in CRC progression remains largely unknown.

NCL (nucleolin) and Sam68 (KHDRBS1) belong to the category of RNA-binding proteins (RBPs), which control RNA metabolism and biogenesis—including RNA synthesis, pre-RNA splicing, RNA processing, ribosomal assembly and maturation—and play multiple roles in cancer development [17, 18]. Nevertheless, it is unclear whether NCL and Sam68 are involved in lncRNA, in particular, whether they function along with CYTOR to regulate CRC progression. In this study, we performed a comprehensive survey of CYTOR expression in CRC samples from online databases and our tissue bank and clarified the roles that CYTOR plays in CRC progression by interacting with NCL and Sam68.

## Methods

### Clinical materials

Two cohorts of clinical samples from the tissue bank in our laboratory were used in this study according to protocols approved by the Internal Review Board of Zhejiang University. A total of 138 pairs of CRC tissues and matched normal tissues were collected to measure CYTOR levels by qRT-PCR. Each experiment was performed at least three times. Another cohort, including 144 CRC FFPE tissue samples, was used for the detection of NCL and Sam68 expression by immunohistochemistry (IHC), and the IHC scores were evaluated by at least two pathologists. The pathological diagnoses of all samples were evaluated by pathologists. All patients with familial adenomatous polyposis, hereditary non-polyposis CRC, or inflammatory bowel disease were excluded. All tissue samples were obtained from colorectal adenocarcinoma patients without any radiotherapy, chemotherapy, or other adjuvant treatment prior to surgery and diagnosis.

### Public databases

The transcriptome expression profiling of CRC and relevant clinical information in this study were identified by searching public databases online. The key words “colon cancer or colorectal cancer and expression profiling by array” were used to search the GEO databases. In addition, the data from the TCGA database were also included in our study to ensure that the data on CYTOR expression in CRC were not missed.

### Statistical analysis

Data were presented as the mean ± standard deviation. The statistical analyses were conducted using the programs GraphPad (GraphPad Software, San Diego, CA, USA) and SPSS 20.0 (SPSS Inc., Chicago, IL, US), and *p* < 0.05 was considered statistically significant. Student’s t-test was used to test for significant differences between groups. Two-tailed tests were applied to all data if not specified. Kaplan-Meier survival and correlation analysis were performed in SPSS 20.0.

### Experimental procedures

CRC cell lines were cultured and transfected with either CYTOR shRNA or overexpression vectors. The proliferation and metastasis capacity of the cells were assessed by CCK-8, colony assays and transwell migration/invasion assays in vitro and by subcutaneous tumor growth in nude mice or mouse tail-vein injection assays in vivo. A revised ChIRP protocol and mass spectrometry (MS) were used to pull down and identify CYTOR RNA-binding proteins. RNA immunoprecipitation (RIP) was carried out to verify the interaction between CYTOR and its binding proteins. CYTOR EXON-1-deleted and EXON-4-deleted cells were constructed by combining CRISPR/Cas9 and the donor vector. Co-immunoprecipitation and RIP were performed to confirm the interaction motif of CYTOR. A luciferase reporter system and an immunoblotting assay were used to investigate the downstream regulatory targets and interacting proteins of CYTOR.

### Additional methods

All further information can be found in the Additional file 1 section.

## Results

### Overexpression of CYTOR in CRC is associated with poor prognosis

**Fig. 1:**
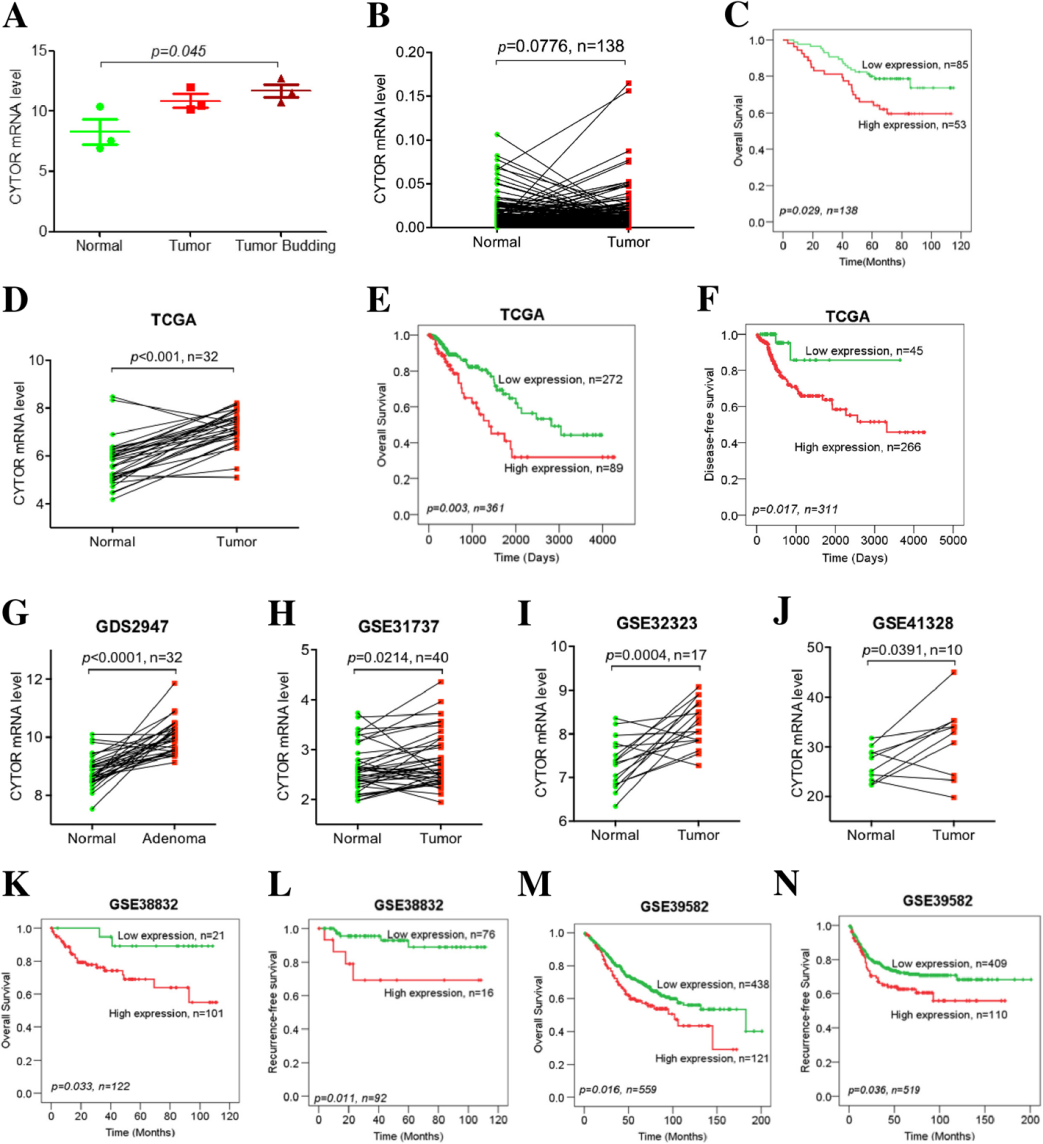
CYTOR up-regulation in CRC samples with poor outcome. **a** Up-regulation of CYTOR in laser-microdissection-captured tumor budding cells, tumor cells compared with normal epithelial cells, *n* = 3 (CYTOR level represents the fluorescence signal value from microarray). **b** Higher expression of CYTOR in CRC samples than the matched normal tissues from our tissue bank, measured by qRT-PCR. **c** Kaplan-Meier plots of overall survival for CRC samples from our tissue bank, higher CYTOR expression with poorer survival. **d** Higher expression of CYTOR in CRC samples than the matched normal tissues from the TCGA database. **e** Kaplan-Meier plots of overall survival and **f** disease-free survival for CRC samples from the TCGA database, higher CYTOR expression with poorer survival. **g** Higher expression of CYTOR in CRC samples than the matched normal tissues from the GDS2947, **h** GSE31737, **i** GSE32323 and **j** GSE41328 databases. **k** Kaplan-Meier plots of overall survival and **l** recurrence-free survival for CRC samples from the GSE38832 database, higher CYTOR expression with poorer survival. **m** Kaplan-Meier plots of overall survival and **n** recurrence-free survival for CRC samples from the GSE39582 database, higher CYTOR expression with poorer survival

Our previous expression profile data [11] showed that CYTOR was up-regulated in CRC and tumor budding cells (Fig. 1a). We further examined the expression of CYTOR in 138 pairs of matched CRC and normal tissues by qRT-PCR, which revealed that CYTOR was up-regulated in CRC tissues (Fig. 1b). Moreover, there was a significant correlation between CYTOR expression and overall patient survival, and patients with higher CYTOR expression showed worse clinical outcome (Fig. 1c). To validate our results, we evaluated the expression levels of CYTOR in the matched pairs of CRC and normal tissue samples using the TCGA RNA-seq database (Fig. 1d), GEO GDS2947 database (Fig. 1g), GEO GSE31737 database (Fig. 1h), GEO GSE32323 database (Fig. 1i) and GEO GSE41328 database (Fig. 1j). Consistent with our observation, the results from these datasets showed significantly up-regulated CYTOR expression in tumor tissue samples. More interestingly, overall survival data showed a poorer prognosis with higher CYTOR levels in the TCGA database (Fig. 1e), GEO GSE38832 database (Fig. 1k), GEO GSE39582 database (Fig. 1m), GEO GSE17536 database (Additional file 2: Figure S1A), GEO GSE17537 database (Additional file 2: Figure S1C), and GEO GSE29621 database (Additional file 2: Figure S1H). Similarly, the expression of CYTOR in CRC was also negatively correlated with disease- or recurrence-free survival in the TCGA database (Fig. 1f), GEO GSE38832 database (Fig. 1l), GEO GSE39582 database (Fig. 1n), GEO GSE17536 database (Additional file 2: Figure S1B), GEO GSE24549-GPL5175 database (Additional file 2: Figure S1J), and GEO GSE24550-GPL11028 database (Additional file 2: Figure S1K).

However, no such correlation was observed between CYTOR and overall, disease-free or recurrence-free survival in some other databases, such as GEO GSE17537 (Additional file 2: Figure S1D), GEO GSE56699 (Additional file 2: Figure S1E and F), GEO GSE16125 (Additional file 2: Figure S1G), GEO GSE24549-GPL11028 (Additional file 2: Figure S1I), GEO GSE24550-GPL5175 (Additional file 2: Figure S1L), GEO GSE31595 (Additional file 2: Figure S1M) and GEO GSE33113 (Additional file 2: Figure S1N). To address this issue, we performed a meta-analysis to systematically evaluate the association between CYTOR and CRC survival risk, combining our data with all the other online CYTOR data. When the cutoff value of CYTOR expression was set according to the receiver operating characteristic (ROC) curve, the patients with higher expression of CYTOR had significantly poorer overall survival (OS; pooled HR, 1.86; 95% CI, 1.50–2.30; Additional file 3: Figure S2A) and disease- or recurrence-free survival (DFS; pooled HR, 1.67, 95%CI, 1.33–2.09; Additional file 3: Figure S2B). When the cutoff value of CYTOR expression was set as P50, CYTOR expression was also negatively associated with overall survival (OS; pooled HR, 1.22; 95% CI, 1.01–1.48; Additional file 3: Figure S2C) and disease- or recurrence-free survival (DFS; pooled HR, 1.30, 95%CI, 1.07–1.58; Additional file 3: Figure S2D). Funnel plots further showed no bias among these databases, which confirmed that CYTOR was a risk factor for survival in colorectal cancer (Additional file 4: Figure S3). Taken together, these clinical data revealed the strong association between CYTOR expression and CRC development/prognosis.

### CYTOR promotes CRC progression in vitro and in mouse xenografts

**Fig. 2:**
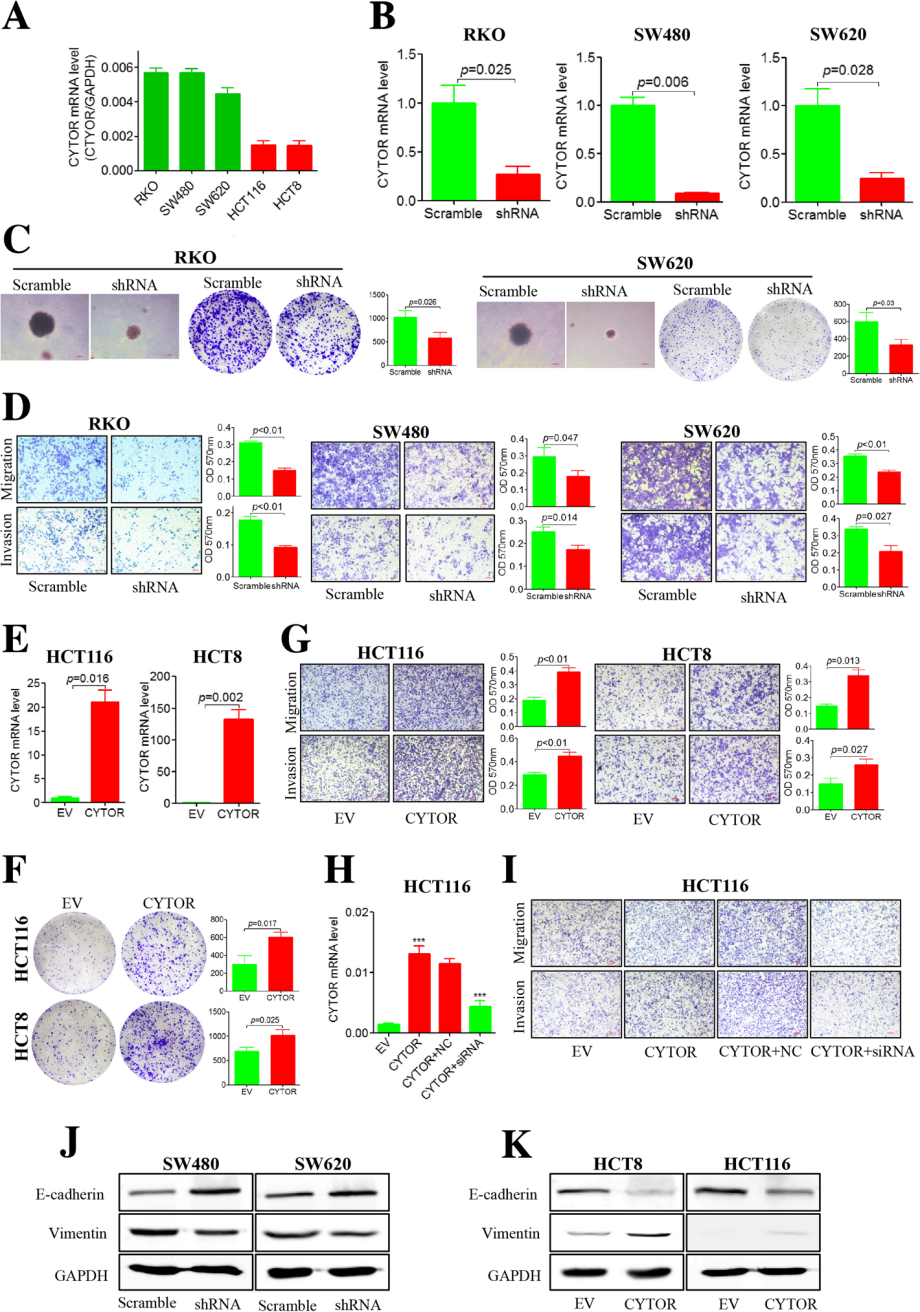
CYTOR promotes anchorage-independent growth and migration/invasion in vitro*.* **a** CYTOR levels in RKO, SW480, SW620, HCT116 and HCT8 cell lines by qRT-PCR. **b** Knockdown of CYTOR by shRNA in the RKO, SW480 and SW620 cell lines. **c** Reduction of colony formation ability for CYTOR knockdown (shRNA) RKO and SW620 cells compared with control (scramble) by soft agar and plates assay (right histogram represents quantification analysis). **d** Decrease of migration/invasive potential for CYTOR knockdown RKO, SW480 and SW620 cells compared with control by transwell assay (right histogram represents quantification analysis). **e** qRT-PCR for CYTOR levels in empty-vector (EV) and overexpression-vector (CYTOR)-transfected HCT116 and HCT8 cells. **f** Increase of colony formation ability for overexpression-vector (CYTOR)- transfected HCT116 and HCT8 cells compared with empty-vector (EV) by plates colony formation assay (Right histogram represents quantification analysis). **g** Increase of migration/invasive potential for CYTOR overexpression (CYTOR) HCT116 and HCT8 cells compared with empty-vector (EV) by transwell assay (Right histogram represents quantification analysis). **h** qRT-PCR for CYTOR levels in HCT116 cells with a CYTOR overexpression vector (CYTOR) and co-transfection CYTOR siRNA (CYTOR+siRNA). **i** Increase of migration/invasive potential for CYTOR-overexpressing (CYTOR, Lane 2) HCT116 cells and rescue potential of CYTOR siRNA (CYTOR+siRNA, Lane 4) by transwell assay. **j** Expression of E-cadherin and Vimentin in CYTOR knockdown SW480 and SW620 cells by immunoblotting. **k** Expression of E-cadherin and Vimentin in CYTOR-overexpressing HCT116 and HCT8 cells by immunoblotting

Next, we examined the CYTOR levels in CRC cell lines, and higher expression was found in RKO, SW480 and SW620 cells than in HCT116 or HCT8 cells (Fig. 2a). When we stably knocked down CYTOR in RKO, SW480 and SW620 cells (Fig. 2b), the colony-forming potential of RKO and SW620 was inhibited (Fig. 2c), and no colonies of SW480 cells were observed. In addition, shRNA-mediated CYTOR knockdown significantly decreased migration and invasion compared with the scramble control in these cell lines (Fig. 2d). To avoid off-target effects of shRNA, we designed and synthesized another two siRNAs to knockdown CYTOR in RKO, SW480 and SW620 cells (Additional file 5: Figure S4A). The results showed knockdown of CYTOR by siRNAs also inhibited the potential of colony-forming (Additional file 5: Figure S4B), migration and invasion in RKO (Additional file 5: Figure S4C), SW480 (Additional file 5: Figure S4D) and SW620 (Additional file 5: Figure S4E) cell lines, which were consistent with the results from shRNA. Furthermore, ectopic expression of CYTOR in the HCT116 and HCT8 cell lines (Fig. 2e) not only promoted colony formation (Fig. 2f) but also enhanced the capacity for migration and invasion (Fig. 2g). More interestingly, when the ectopic expression of CYTOR was inhibited by specific siRNAs in the transfected HCT116 cells (Fig. 2h), the enhanced migration and invasion were also decreased by these siRNAs (Fig. 2i). These results demonstrated that CYTOR could promote the anchorage-independent growth, migration and invasion of CRC cells in vitro*.* As tumor budding cells are considered a histological phenomenon of EMT, which contributes to tumor metastasis [19–21], we also evaluated the relationship between CYTOR and EMT markers. In SW480 and SW620 cells, knockdown of CYTOR increased E-cadherin expression while decreasing Vimentin expression (Fig. 2j). On the other hand, overexpression of CYTOR inhibited E-cadherin expression while increasing Vimentin expression in HCT8 and HCT116 cells (Fig. 2k). Analysis of the current GEO database (GSE29621 and GSE38832) showed that CYTOR expression was negatively correlated with the epithelial marker E-cadherin and positively correlated with mesenchymal markers including Vimentin, N-cadherin, FN1, Twist, and MMP9 (Additional file 6: Figure S5).

**Fig. 3:**
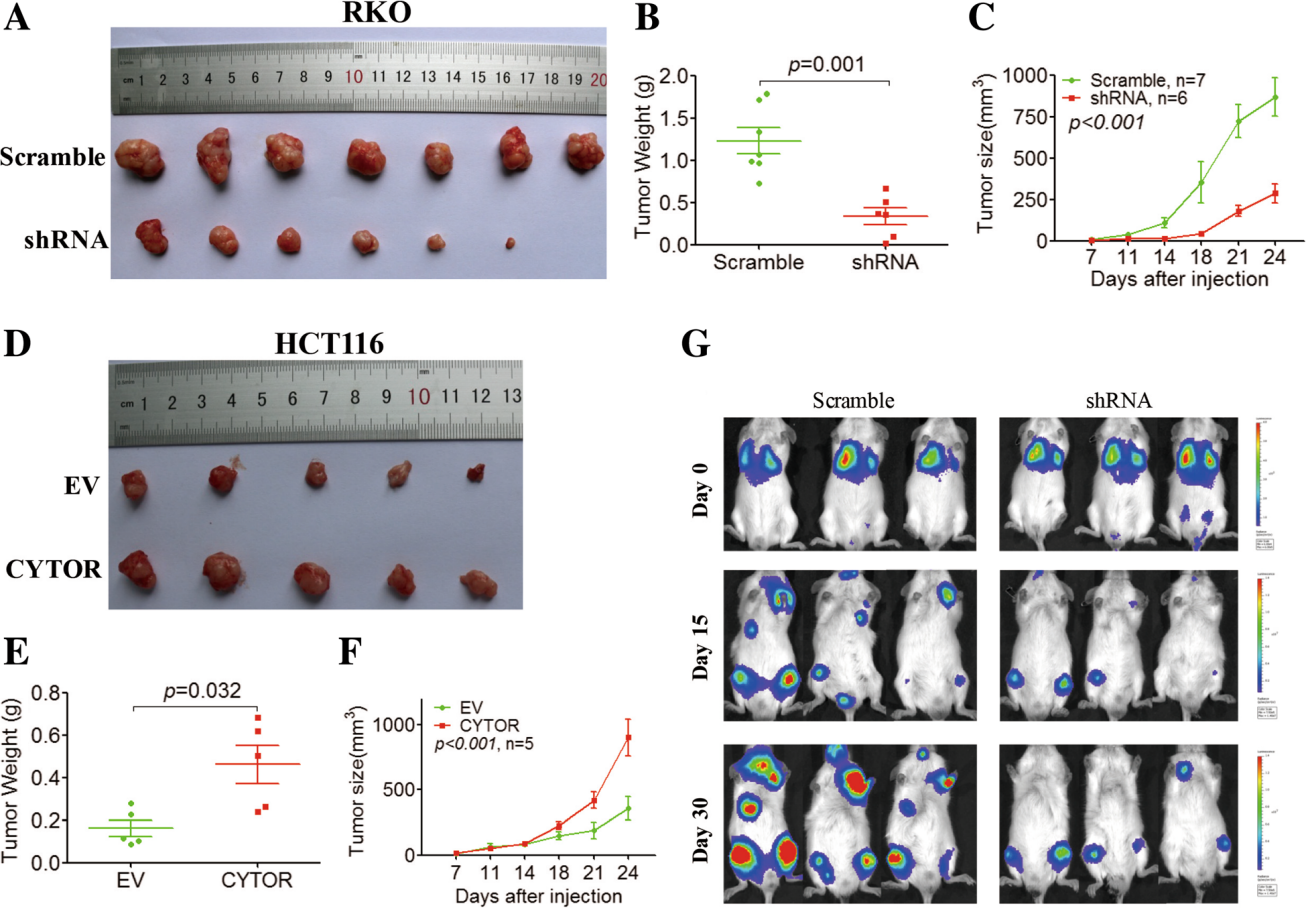
CYTOR promotes tumor growth and metastasis in mouse xenografts. **a** Volume of Xenograft tumors from BALB/c-nu/nu male mice subcutaneously injected with CYTOR knockdown (shRNA) RKO cells and control (scramble). **b** Xenograft tumor weight for CYTOR knockdown (shRNA) RKO cells and control (scramble) in the xenograft model. **c** Tumor growth curves of CYTOR knockdown (shRNA) RKO cells and control (scramble) and in the xenograft model. **d** Volume of Xenograft tumors from BALB/c-nu/nu male mice subcutaneously injected with control (EV) or CYTOR-overexpressing (CYTOR) HCT116 cells. **e** Xenograft tumor weight for CYTOR-overexpressing (CYTOR) HCT116 cells and control (EV) in the xenograft model. **f** Tumor growth curves of control (EV) and CYTOR-overexpressing (CYTOR) HCT116 cells in the xenograft model. **g** Representative images of luciferase signals in pulmonary metastatic luciferase foci after tail-vein injection of control (scramble) or CYTOR knockdown (CYTOR shRNA) RKO cells in immunodeficient mice

We also investigated whether CYTOR was functionally involved in CRC progression in mouse xenografts. When RKO cells transfected with CYTOR shRNA were subcutaneously injected into nude mice, the volume (Fig. 3a) and weight (Fig. 3b) of the xenograft tumors were significantly decreased compared with the scramble control group. Moreover, tumor growth was also repressed by CYTOR knockdown as shown in the tumor growth curve (Fig. 3c). By contrast, overexpression of CYTOR increased the volume (Fig. 3d) and weight (Fig. 3e) of the xenograft tumors and promoted tumor growth in vivo (Fig. 3f). To explore whether CYTOR also promotes CRC metastasis in vivo, we intravenously injected luciferase-labeled control or CYTOR knockdown RKO cells into NOD/SCID mice and subjected them to bioluminescent imaging to monitor metastasis. The results showed that the whole-body luminescence signals in the CYTOR knockdown group were significantly reduced compared with those of the control group after 30 days (Fig. 3g). Overall, our data indicated that CYTOR could promote EMT and CRC progression.

### CYTOR mediates complex formation between NCL and Sam68

**Fig. 4:**
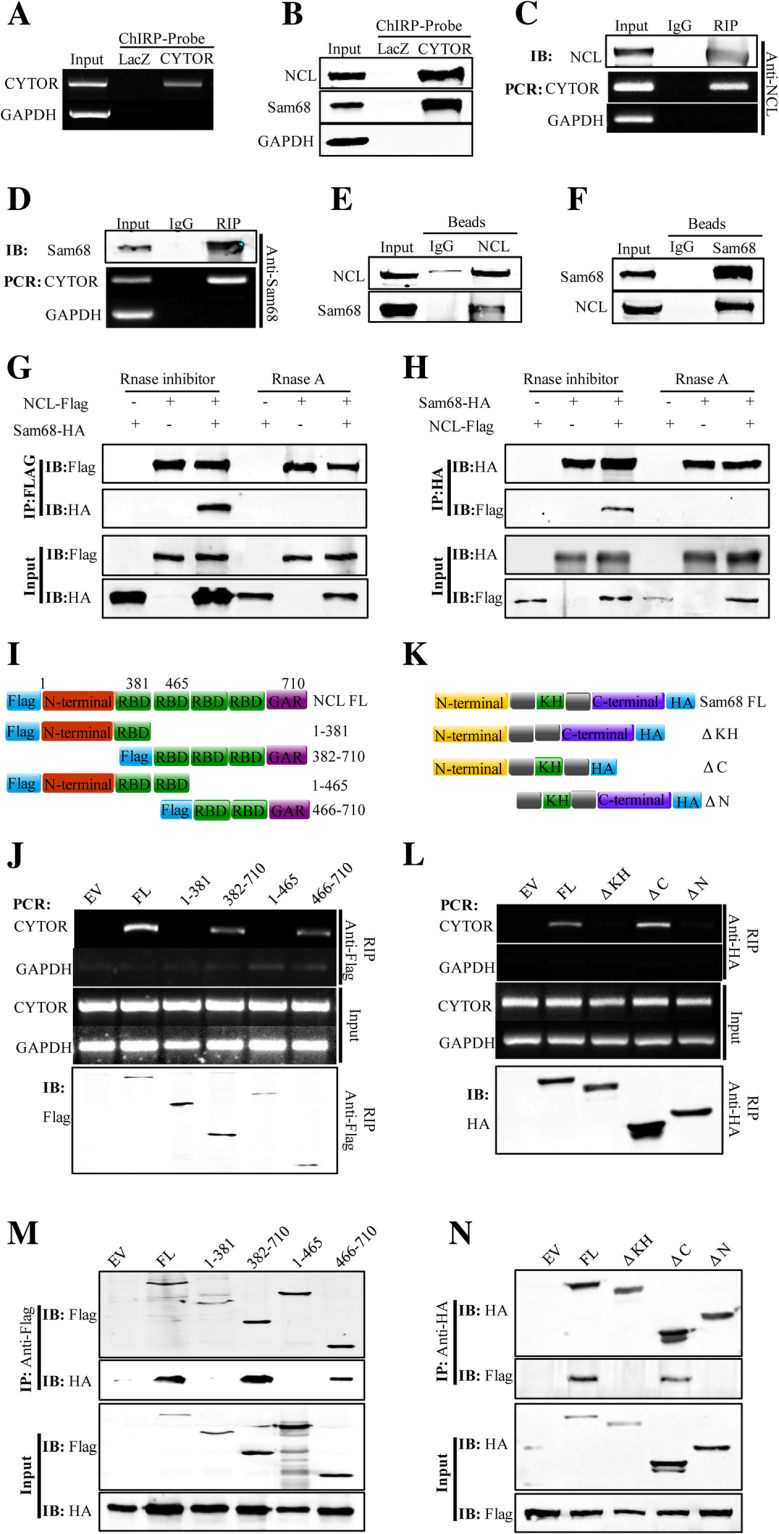
CYTOR mediates complex formation between NCL and Sam68. **a** RT-PCR for detection of CYTOR and GAPDH pull down by ChIRP probes of LacZ and CYTOR in RKO cells. **b** Immunoblotting for detection of NCL and Sam68 pull down by ChIRP probes of LacZ and CYTOR in RKO cells. **c** Immunoblotting for detection of NCL (upper panel) and RT-PCR for detection of CYTOR and GAPDH (lower panels) by RIP with antibody against NCL in RKO cells. **d** Immunoblotting for detection of Sam68 (upper panel) and RT-PCR for detection of CYTOR and GAPDH (lower panels) by RIP with antibody against Sam68 in RKO cells. **e** Immunoblotting for detection of endogenous NCL and Sam68 immunoprecipitated by NCL-specific antibody in RKO cells. **f** Immunoblotting for detection of endogenous Sam68 and NCL immunoprecipitated by Sam68-specific antibody in RKO cells. **g** Immunoblotting for detection of exogenous FLAG-tagged NCL and HA-tagged Sam68 immunoprecipitated by anti-FLAG antibody with or without RNaseA treatment in 293 T cells. **h** Immunoblotting for detection of exogenous HA-tagged Sam68 and FLAG-tagged NCL immunoprecipitated by anti-HA antibody with or without RNaseA treatment in 293 T cells. **i** Schematic of the FLAG-tagged full-length and truncation mutant constructs of NCL. **j** RT-PCR for detection of CYTOR and GAPDH (upper panel) and immunoblotting for detection of truncation mutant NCL with anti-FLAG (lower panels) by RIP with anti-FLAG antibody in 293 T cells with different truncation mutant constructs of NCL. **k** Schematic of the HA-tagged full-length and truncation mutant constructs of Sam68. **l** RT-PCR for detection of CYTOR and GAPDH (upper panel) and immunoblotting for detection of truncation mutant Sam68 with anti-HA (lower panels) by RIP with anti-HA antibody in 293 T cells with different truncation mutant constructs of Sam68. **m** Immunoblotting for detection of exogenous HA-tagged Sam68 and different FLAG-tagged truncation mutants of NCL immunoprecipitated by anti-FLAG antibody in 293 T cells. **n** Immunoblotting for detection of exogenous FLAG-tagged NCL and different HA-tagged truncation mutants of Sam68 immunoprecipitated by by anti-HA antibody in 293 T cells

To explore the detailed mechanism whereby CYTOR regulates CRC progression, we first investigated the distribution of CTYOR in RKO cells and found it distributed in both cytoplasm and nucleus (Additional file 7: Figure S6A). Then, we designed a set of specific probes labeled with biotin to pull down the proteins that directly bind to CYTOR [8, 22]. The efficiency and specificity of the probes were confirmed by PCR (Fig. 4a). We isolated the proteins from the pull-down complex by SDS-PAGE electrophoresis (Additional file 7: Figure S6B) and identified NCL and Sam68 as the CYTOR-binding proteins by MS assays (Additional file 7: Figure S6C) in RKO cells. Furthermore, the interactions between CYTOR and NCL, CYTOR and Sam68 were confirmed by immunoblotting (Fig. 4b). In addition, RIP assays revealed that both NCL (Fig. 4c) and Sam68 (Fig. 4d) could pull down CYTOR directly.

The results above demonstrated that NCL and Sam68 could bind to CYTOR; next, we investigated whether a complex could be formed between NCL and Sam68. The co-IP assay showed that NCL was able not only to pull down Sam68 (Fig. 4e) but also to be immunoprecipitated by Sam68 (Fig. 4f) in live cells. Then, we simultaneously transfected the FLAG-tagged NCL (NCL-FLAG) and HA-tagged Sam68 (Sam68-HA) expression vectors into 293 T cells, and the harvested cell lysates were treated with either RNase inhibitor or RNaseA, followed by dual co-IP assays with either anti-FLAG or anti-HA antibody. The results showed that NCL and Sam68 protein could be co-immunoprecipitated reciprocally in the RNase-inhibitor-treated samples (Fig. 4g and h, 3rd lanes) but not in RNaseA-treated samples (Fig. 4g and h, 6th lanes). These results suggested that the interaction between NCL and Sam68 depended on the existence of RNA.

The NCL protein consists of six main domains, including the N-terminal domain, four RNA binding domains (RBDs) and the GAR domain [23]. To further identify the sites on NCL that interact with CYTOR, we constructed four deletion mutants of FLAG-tagged NCL (Fig. 4i). It was shown that in addition to full-length NCL (FL), the mutants that included the 3rd and 4th RBDs and the GAR domain could pull down CYTOR (Fig. 4j), indicating that these domains might be the key sites of interaction with CYTOR. Moreover, we also constructed KH-domain-deleted (ΔKH), N-terminus-deleted (ΔN) and C-terminus-deleted (ΔC) HA-tagged Sam68 expression vectors [24] (Fig. 4k). RIP results revealed that the ΔC mutant could bind CYTOR, while the ΔKH and ΔN mutants could not (Fig. 4l), demonstrating that the KH and N-terminal domains were the sites of interaction with CYTOR. More intriguingly, co-IP assays revealed that the 3rd and 4th RBDs and the GAR domain of NCL were required for binding to Sam68 (Fig. 4m), and the KH and N-terminal domains of Sam68 were indispensable to its interaction with NCL (Fig. 4n). Taken together, the above data indicated that CYTOR plays an essential part in forming a complex between NCL and Sam68 by interacting with specific motifs on the two proteins.

### EXON1 of CYTOR is important for its biological function of forming a complex with NCL and Sam68

**Fig. 5:**
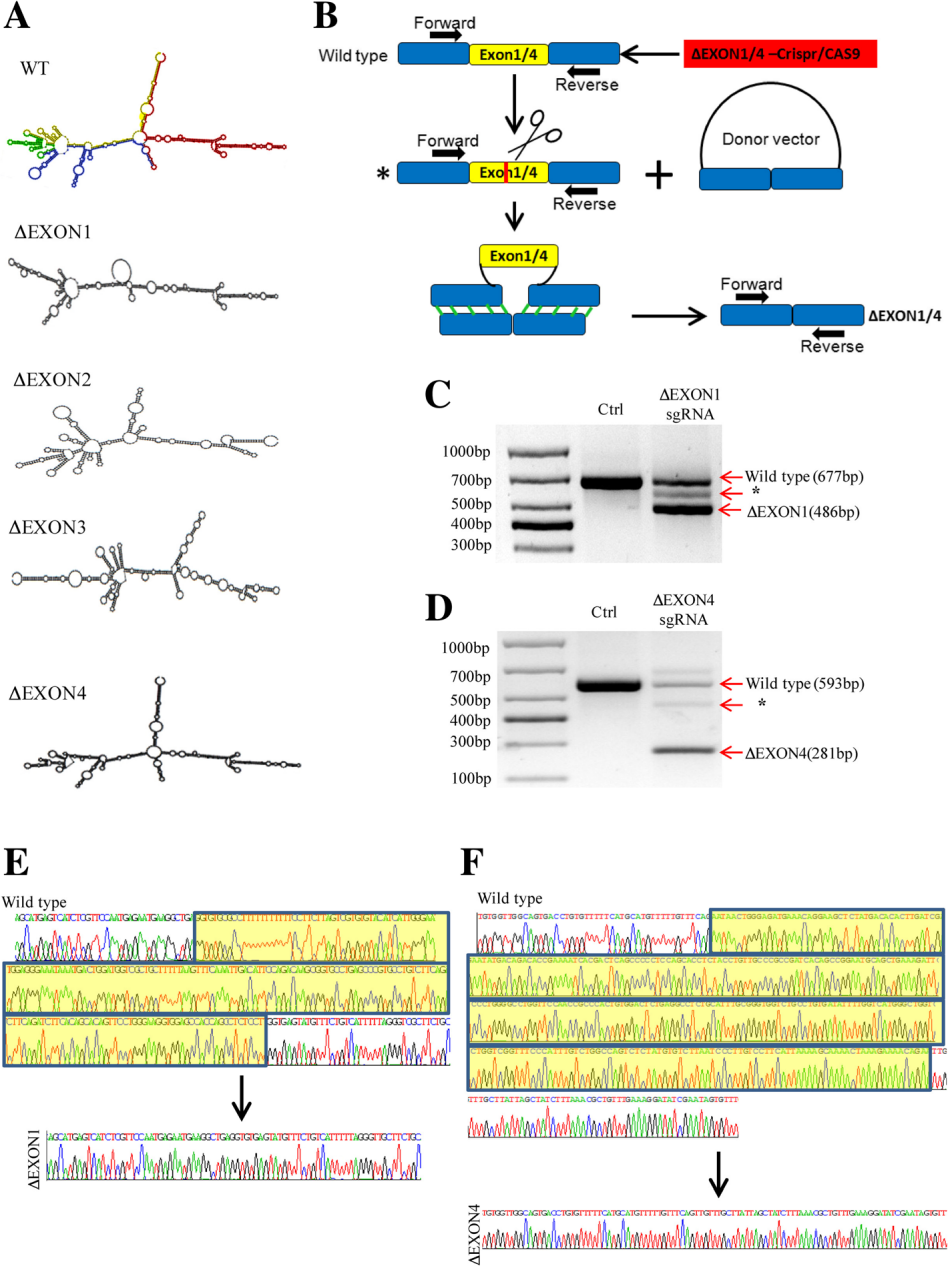
Production of CYTOR mutant cells by CRISPR/Cas9. **a** Prediction of secondary RNA structure of wild-type (WT) CYTOR and various exon-deleted CYTOR mutants. **b** Schematic diagram for CRISPR/Cas9 and donor vector to delete EXON1 or EXON4 of CYTOR. **c** RT-PCR and gel electrophoresis to assess the editing efficiency of CRISPR-sgRNA specific to EXON 1. *indicates Non-homologous end joining with an Indel. **d** RT-PCR and gel electrophoresis to assess the editing efficiency of CRISPR-sgRNA specific to EXON 4. *indicates Non-homologous end joining with an Indel. **e** DNA sequencing to identify the exon1-deleted RKO cells (ΔEXON1) through clone screening. **f** DNA sequencing to identify the exon4-deleted RKO cells (ΔEXON4) through clone screening

**Fig. 6:**
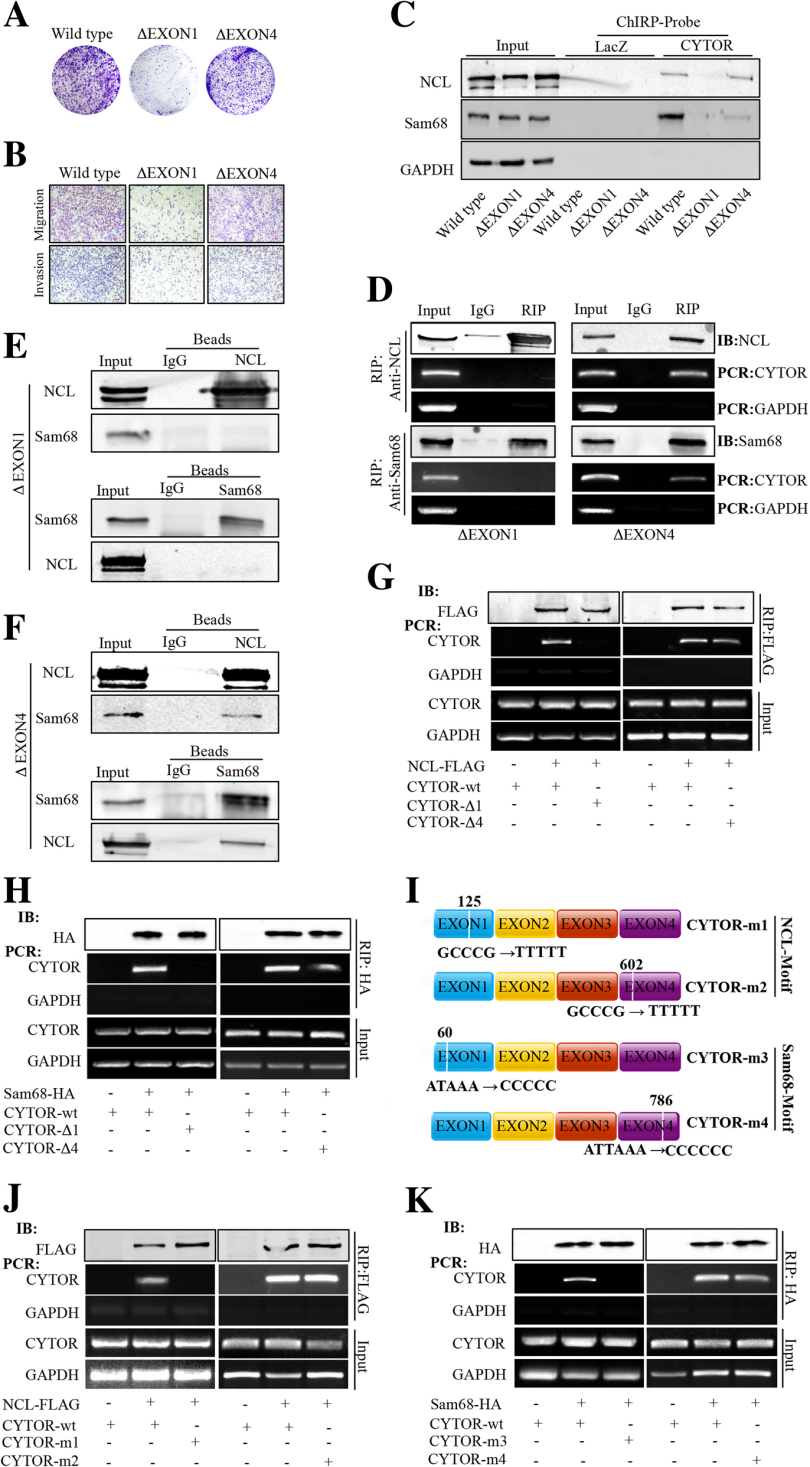
EXON1 is a key functional site of CYTOR and mediates the interaction between NCL and Sam68. **a** Decrease of colony formation ability for EXON1-deleted mutant (ΔEXON1) and EXON4-deleted mutant (ΔEXON4) RKO cells by CRISPR/Cas9 compared with wild-type. **b** Decrease of migration/invasive potential for EXON1-deleted mutant (ΔEXON1) and EXON4-deleted mutant (ΔEXON4) RKO cells compared with wild-type. **c** Immunoblotting for detection of NCL and Sam68 pull down by ChIRP probes of LacZ and CYTOR in wild-type, EXON1-deleted mutant (ΔEXON1) and EXON4-deleted mutant (ΔEXON4) RKO cells. **d** Immunoblotting for detection of NCL and Sam68 and RT-PCR for detection of CYTOR and GAPDH by RIP with antibodies against NCL and Sam68 in EXON1-deleted mutant (ΔEXON1) and EXON4-deleted mutant (ΔEXON4) RKO cells. **e** Reciprocal immunoprecipitation between endogenous Sam68 and NCL with separate specific antibodies in EXON1-deleted mutant (ΔEXON1) RKO cells. **f** Reciprocal immunoprecipitation between endogenous Sam68 and NCL with separate specific antibodies in EXON4-deleted mutant (ΔEXON4) RKO cells. **g** RT-PCR for detection of CYTOR and GAPDH by RIP with anti-FLAG in 293 T cells co-transfected with FLAG-tagged NCL and wild-type (CYTOR-wt), EXON1-deleted mutant (CYTOR-Δ1) or EXON4-deleted mutant (CYTOR-Δ4) CYTOR. **h** RT-PCR for detection of CYTOR and GAPDH by RIP with anti-HA in 293 T cells co-transfected with HA-tagged Sam68 and wild-type, EXON1-deleted mutant or EXON4-deleted mutant CYTOR. **i** Schematic of CYTOR motif mutant constructs; CYTOR-m1, mutant for the NCL-specific motif in EXON1 of CYTOR; CYTOR-m2, mutant for the NCL-specific motif in EXON4 of CYTOR; CYTOR-m3, mutant for the Sam68-specific motif in EXON1 of CYTOR; CYTOR-m4, mutant for the Sam68-specific motif of Sam68 in EXON4 of CYTOR. **j** RT-PCR for detection of CYTOR and GAPDH by RIP with anti-FLAG in 293 T cells co-transfected with FLAG-tagged NCL and wild-type CYTOR, CYTOR-m1 and CYTOR-m2. **k** RT-PCR for detection of CYTOR and GAPDH by RIP with anti-HA in 293 T cells co-transfected with HA-tagged Sam68 and wild-type CYTOR, CYTOR-m3 and CYTOR-m4

We next intended to identify the motifs in CYTOR that are essential for its interaction with NCL and Sam68. The secondary structure of CYTOR was predicted using the RNA fold web server [25]. It was found that only the EXON1 deletion (ΔEXON1) and not the other EXON deletion mutants (ΔEXON2, ΔEXON3 and ΔEXON4) of CYTOR could induce a dramatic change in the secondary structure relative to wild-type (WT) CYTOR (Fig. 5a). Thus, we used the CRISPR/Cas9 method (Fig. 5b) to construct ΔEXON1 (Fig. 5c) and ΔEXON4 RKO cells (Fig. 5d). The mutant cells were screened and confirmed as homozygous EXON1-deleted (Fig. 5e) and EXON4-deleted (Fig. 5f) clones by sequencing. As shown in Fig. 6a, the colony-forming potential of ΔEXON1 cells was reduced, but no changes were observed in ΔEXON4 cells. Similarly, deleting EXON1 of CYTOR inhibited cell migration and invasion, but deleting EXON4 had no such effect (Fig. 6b). Interestingly, a ChIRP assay showed that CYTOR-specific probes could pull down NCL and Sam68 in wild-type and ΔEXON4 RKO cells but not in ΔEXON1 RKO cells (Fig. 6c). Additionally, an RIP assay was performed to investigate the endogenous interactions between NCL, Sam68 and CYTOR in these mutant cells, and the results showed that while both NCL and Sam68 could bind to CYTOR in ΔEXON4 RKO cells, neither could pull down CYTOR in ΔEXON1 RKO cells (Fig. 6d). To determine whether complex formation between NCL and Sam68 also requires EXON1 of CYTOR, we evaluated their interaction in ∆EXON1 and ∆EXON4 cells using co-IP. The results showed that while NCL and Sam68 could be immunoprecipitated reciprocally in ∆EXON4 cells (Fig. 6f), no such interaction could be observed in ∆EXON1 cells (Fig. 6e).

To further verify the above results, we co-transfected 293 T cells with exogenous expression vectors for FLAG-tagged NCL, CYTOR EXON1-deletion (CYTOR-Δ1) or EXON4-deletion (CYTOR-Δ4) mutants. An RIP assay with anti-FLAG antibody revealed that NCL could bind to CYTOR-∆4 but not CYTOR-∆1 (Fig. 6g). Similarly, HA-tagged Sam68 could also pull down CYTOR-∆4 but not CYTOR-∆1 (Fig. 6h). Fortunately, we found two NCL-specific binding motifs [26] at the 125th nt of EXON1 and the 602nd nt of EXON4 in the CYTOR RNA sequence, as well as two Sam68-specific binding motifs [27] at the 60th nt of EXON1 and the 786th nt of EXON4 (Fig. 6i). Therefore, we constructed a series of CYTOR expression vectors with mutations in these sites (Fig. 6i). When FLAG-tagged NCL and CYTOR mutants with mutations in the NCL-specific sites (CYTOR-m1 for mutation in EXON1 or CYTOR-m2 for mutation in EXON4) were co-transfected into 293 T cells, an RIP assay showed that NCL could bind to CYTOR-m2 but not to CYTOR-m1 (Fig. 6j). Similarly, while HA-tagged Sam68 could not pull down the CYTOR mutant with the mutation in its specific-binding site on EXON1 (CYTOR-m3), it could bind to the CYTOR mutant with the mutation on EXON4 (CYTOR-m4) (Fig. 6k). Taken together, these data indicated that the formation of heterotrimeric complex of NCL, Sam68, and CYTOR requires their specific interacting sites in CYTOR EXON1.

### NCL and Sam68 act as oncogenes to promote CRC progression

**Fig. 7:**
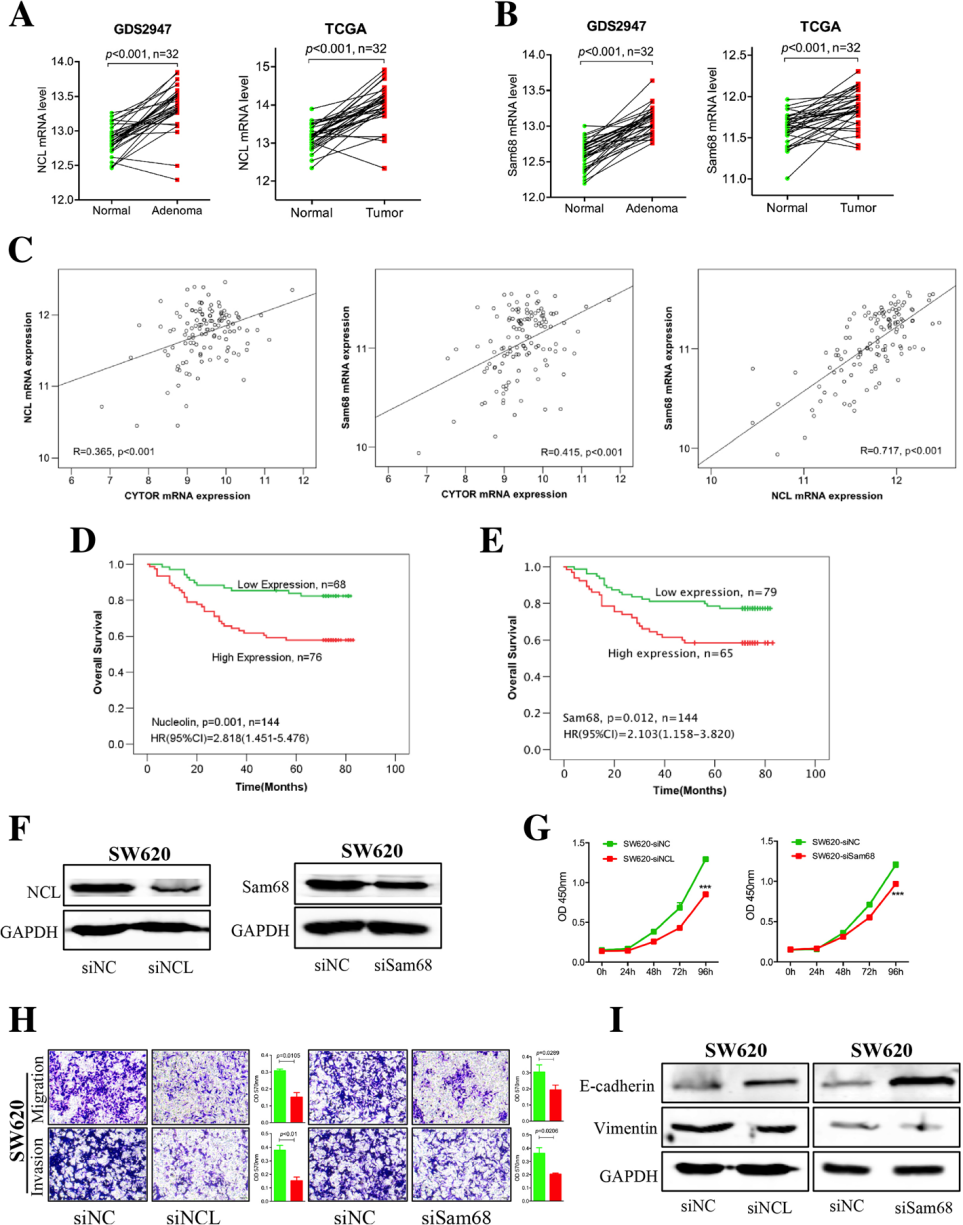
NCL and Sam68 acted as oncogenes and promoted CRC progression. **a** Higher expression of NCL in colorectal cancer than paired matched normal tissue samples from the GDS2947 and TCGA databases. **b** Higher expression of Sam68 in colorectal cancer than paired matched normal tissue samples from the GDS2947 and TCGA databases. **c** Positive correlation between CYTOR, NCL and Sam68 expression in the GSE38832 database. **d** Kaplan-Meier plots of overall survival versus NCL in CRC samples from our tissue bank, higher NCL expression with poorer survival. **e** Kaplan-Meier plots of overall survival versus Sam68 in CRC samples from our tissue bank, higher Sam68 expression with poorer survival. **f** Immunoblotting for detection of NCL and Sam68 in SW620 cells knocked down by siRNAs of NCL and Sam68. **g** Decrease of the proliferation ability for NCL knockdown (siNCL) and Sam68 knockdown (siSam68) SW620 cells compared with control (siNC) by CCK-8. **h** Decrease of migration/invasive potentials for NCL knockdown (siNCL) and Sam68 knockdown (siSam68) SW620 cells compared with control (siNC) by Transwell assay. **i** Expression of E-cadherin and Vimentin in NCL and Sam68 knockdown SW620 cells by immunoblotting

Although it was confirmed that NCL and Sam68 could directly interact with CYTOR to form a complex, their function in CRC progression was still unknown. The results from online databases showed that NCL (Fig. 7a and Additional file 8: Figure S7A) and Sam68 (Fig. 7b and Additional file 8: Figure S7B) were both up-regulated in tumor samples compared with paired normal samples. In addition, the expression levels of CYTOR, NCL, and Sam68 were positively correlated (Fig. 7c). Our IHC results also revealed that higher NCL (Fig. 7d) and Sam68 (Fig. 7e) expression levels were associated with poorer survival rates. These results suggest that both NCL and Sam68 are involved in CRC progression.

In an effort to decipher the detailed functions of both proteins, we knocked down NCL or Sam68 using siRNAs (Fig. 7f), and it was shown that the proliferation of SW620 (Fig. 7g) and RKO (Additional file 8: Figure S7C) cells was inhibited under those conditions. Consistently with the CYTOR knockdown results, loss of NCL and Sam68 also repressed the migration and invasion activity of SW620 (Fig. 7h) and RKO (Additional file 8: Figure S7D) cells. Furthermore, the epithelial marker E-cadherin was increased and the mesenchymal marker Vimentin was decreased by NCL and Sam68 siRNAs (Fig. 7i). To verify these observations, we investigated the relationship between the expression of NCL/Sam68 and EMT markers in the GSE38832 database, and the results showed that both NCL and Sam68 expression were negatively correlated with the epithelial marker E-cadherin and positively correlated with the mesenchymal marker Vimentin, N-cadherin, Snail, and Twist (Additional file 9: Figure S8A for NCL and Additional file 9: Figure S8B for Sam68). Collectively, NCL and Sam68 may function as oncogenes to promote CRC EMT and progression.

### The NCL-CYTOR-Sam68 complex activates the NF-κB pathway

**Fig. 8:**
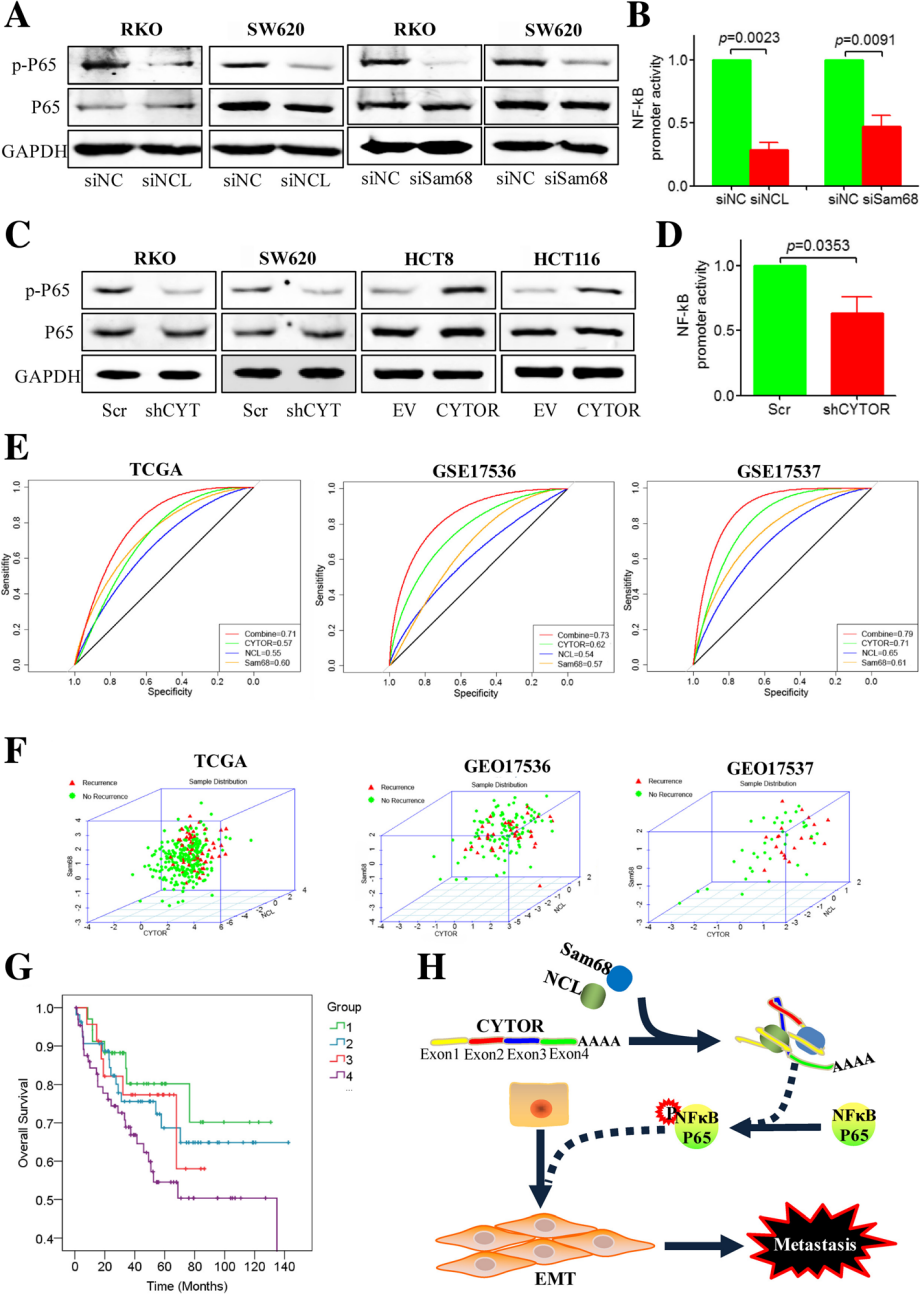
The NCL-CYTOR-Sam68 complex activated the NF-κB pathway and acted as a prognostic biomarker. **a** Expression of P65 and phosphorylated P65 in NCL and Sam68 knockdown RKO and SW620 cells by immunoblotting. **b** Luciferase promoter reporter assays of NF-κB in NCL and Sam68 knockdown cells. **c** Expression of P65 and phosphorylated P65 in CYTOR knockdown cells and in CYTOR-overexpressing HCT8 and HCT116 cells by immunoblotting. **d** Luciferase promoter reporter assays of NF-κB in CYTOR knockdown cells. **e** ROC curve analysis for the combination of CYTOR, NCL and Sam68 in the TCGA, GSE17536 and GSE17537 databases. **f** 3D curve assay for the relationship between recurrence and the expression distribution of CYTOR, NCL and Sam68. **g** Kaplan-Meier plots of overall survival for the combination of CYTOR, NCL and Sam68 in GSE17536 database; group 1 has low expression for all three molecules; group 2 has low expression of CYTOR only; group 3 has high expression of CYTOR only; group 4 has high expression of all three molecules. **h** Schematic model of the function of CYTOR in CRC progression. CYTOR mediates the interaction of NCL and Sam68 through specific motifs in its EXON1 and activates the NF-κB signaling pathway to promote CRC EMT and metastasis

The NF-κB pathway has been recognized as a key player in CRC progression, being involved in the EMT process [28, 29]; therefore, we evaluated the effect of the CYTOR-NCL-Sam68 complex on the NF-κB signaling pathway. The results showed that both NCL and Sam68 knockdown caused a dramatic decrease in phosphorylated P65 in CRC cell lines (Fig. 8a) and attenuated the transcriptional activity of NF-κB as shown by a luciferase promoter assay (Fig. 8b). Similarly, CYTOR knockdown also significantly decreased the level of phosphorylated P65, while overexpression of CYTOR increased the expression of phosphorylated P65 (Fig. 8c). Moreover, a luciferase promoter assay showed decreased NF-κB promoter activity when CYTOR expression was inhibited by shRNA (Fig. 8d). These results demonstrated that the NCL-CYTOR-Sam68 complex might promote CRC progression by activating the NF-κB signaling pathway.

### The NCL-CYTOR-Sam68 complex can be used as a biomarker for CRC prognosis

To further evaluate the clinical significance of the CYTOR-NCL-Sam68 complex, we performed ROC analysis for CYTOR, NCL and Sam68, either separately or combined, in the TCGA, GSE17536 and GSE17537 databases. The results showed that the AUC for combined CYTOR, NCL, and Sam68 was greater than the individual AUC for CYTOR, NCL or Sam68 (Fig. 8e). Interestingly, in the 3D curve assay, the area with high expression of CYTOR, NCL and Sam68 represented samples from recurrent patients (Fig. 8f). The overall survival data from the GSE17536 database also showed that the patients with high expression of all three molecules (group 4) displayed the worst prognosis (Fig. 8g). These clinical data suggest that the NCL-CYTOR-Sam68 complex can be used as a biomarker for CRC recurrence and prognosis.

## Discussion

It has been shown that lncRNAs function primarily through their interactions with cellular macromolecules, such as chromatin DNA, proteins and RNAs [30–32]. In the present study, we elucidated that lncRNA CYTOR forms a trimeric complex with NCL and Sam68 through the specific motifs on EXON1 and activates the NF-κB signaling pathway to promote CRC EMT and progression (Fig. 8h).

Our clinical data also revealed that CYTOR was frequently up-regulated in CRC samples and that its overexpression was significantly associated with poor prognosis for CRC patients. A systematic analysis of online databases suggested that CYTOR could be considered a risk factor for recurrence and prognosis in CRC patients. Our results indicated that CYTOR probably acts as a proto-oncogene in CRC, which is similar to its role in lung cancer [33], gastric cancer [12], and other cancers. Mechanistically, CYTOR might function as a competing endogenous RNA to regulate tumorigenesis and progression. For example, CYTOR sponged miRNA-103a-3p to promote malignant progression of glioma stem cells [34], modulated the expression of miR-193a-3p to confer resistance to oxaliplatin in colon cancer [14], and negatively regulated miR-205 to promote renal cell carcinoma progression [35]. Proteins interacting with CYTOR had been investigated in other cancer types; for example, EZH2 and EGFR were reported to interact with CYTOR in gastric cancer [36, 37]. In our ChIRP and MS assays, EZH2 and EGFR were not detected; a possible explanation might be the different types of cancer cells used.

NCL, one of the two proteins identified, is an acidic phosphoprotein that is abundantly expressed in exponentially growing cells and is located mainly in the nucleolus; it can also be found in the nucleoplasm, cytoplasm, and cell membrane [17]. In addition to ribosome biogenesis, NCL contributes to cancer progression through shuttling between the nucleolus, cytoplasm and cell membrane and regulating BCL-2, P53 and MMP9, and other proteins [38]. Overexpression of NCL was found in several types of cancer, including lung and breast cancer [39]. In particular, ectopic expression of NCL in colorectal cancer was associated with higher aggressiveness and worse prognosis [39], consistent with our results. In this study, we showed that NCL could bind to a specific site in EXON1 of CYTOR, which might contribute to CRC progression and metastasis.

The other protein, SAM68, is a tyrosine-phosphorylated, SRC-associated protein that is present in mitotic cells and plays key roles during cell differentiation and development [18]. Aberrant expression of SAM68 was detected in several types of tumors such as prostate cancer, non-small cell lung cancer, renal cell carcinoma and colorectal cancer, in which high SAM68 expression was inversely associated with overall survival [18, 40]. Our current study also revealed that Sam68 could specifically recognize its binding site in EXON1 of CYTOR and, together with NCL, acted as an oncogene contributing to CRC progression. These data also suggested that EXON1 of CYTOR is the key functional motif that mediates the formation of the heterotrimeric complex.

Dysregulation of NF-κB signaling is a common event in many types of cancer and contributes to tumor initiation and progression by driving the expression of pro-proliferative/anti-apoptotic genes. More importantly, NF-κB signaling also plays critical roles in EMT and cancer progression [29]. In this study, we also demonstrated that the NCL-CYTOR-Sam68 complex could activate the NF-κB signaling pathway, thus promoting EMT and metastasis in CRC. Still, the underlying mechanism whereby CYTOR, NCL and Sam68 regulate the NF-κB signaling pathway needs further investigation.

## Conclusions

We identified the functional roles played in CRC progression by CYTOR, which forms a heterotrimeric complex with NCL and Sam68 through EXON1. We also provided strong clinical evidence for CYTOR as a biomarker of recurrence and prognosis of CRC. In addition, on the basis of the important function of the NCL-CYTOR-Sam68 complex, these molecules might have potential as novel targets for CRC therapy in the future.

## Additional files

Additional file 1: Supplementary Materials and Methods. (DOCX 33 kb)

Additional file 2: **Figure S1.** CYTOR expression and CRC prognosis. (A, B) Kaplan-Meier plots of overall survival (A) and recurrence-free survival (B) for CRC samples from the GSE17536 database. (C, D) Kaplan-Meier plots of overall survival (C) and recurrence-free survival (D) for CRC samples from the GSE17537 database. (E, F) Kaplan-Meier plots of overall survival (E) and recurrence-free survival (F) for CRC samples from the GSE56699 database. (G, H) Kaplan-Meier plots of overall survival for CRC samples from the GSE16125 (G) and GSE29621 (H) databases. (I, J, K, L) Kaplan-Meier plots of disease-free survival for CRC samples from the GSE24549-GPL11028 (I), GSE24549-GPL5175 (J), GSE24550-GPL11028 (K) and GSE24550-GPL5175 (L) databases. (M, N) Kaplan-Meier plots of recurrence-free survival for CRC samples from the GSE31595 (M) and GSE33113 (N) databases. (JPG 688 kb)

Additional file 3: **Figure S2.** A meta-analysis of the association between CYTOR and CRC survival. (A) Forest plots of the association between CYTOR expression and overall survival at the cutoff value set according to the ROC. (B) Forest plots of the association between CYTOR expression and disease- or recurrence-free survival at the cutoff value set according to the ROC. (C) Forest plots of the association between CYTOR expression and overall survival at the P50 cutoff value. (D) Forest plots of the association between CYTOR expression and disease- or recurrence-free survival at the P50 cutoff value. (JPG 1781 kb)

Additional file 4: **Figure S3.** Funnel plots for the relationship between CYTOR and CRC prognosis. (A) Funnel plots of the association between CYTOR expression and overall survival at the cutoff value set according to the ROC. (B) Funnel plots of the association between CYTOR expression and disease- or recurrence-free survival at the cutoff value set by according to the ROC. (C) Funnel plots of the association between CYTOR expression and overall survival at the P50 cutoff value. (D) Funnel plots of the association between CYTOR expression and disease- or recurrence-free survival at the P50 cutoff value. (JPG 1257 kb)

Additional file 5: **Figure S4.** Knockdown of CYTOR inhibited anchorage-independent growth and migration/invasion.(A) qRT-PCR for detection of CYTOR in RKO, SW480 and SW620 cells knocked known by siRNAs of CYTOR. (B) Reduction of colony formation ability for CYTOR knockdown RKO and SW620 cells by siRNAs compared with control (NC). (C, D and E) Decrease of migration/invasive potential for CYTOR knockdown RKO (C), SW480 (D) and SW620 (E) cells by siRNAs compared with control by transwell assay. (JPG 2970 kb)

Additional file 6: **Figure S5.** Correlation analysis between CYTOR and EMT markers. (JPG 567 kb)

Additional file 7: **Figure S6.** CYTOR location in cells and its binding proteins identified by ChIRP and MS. (A) RNA FISH to detection CYTOR location in RKO cells. (B) SDS-PAGE for protein isolation by ChIRP with CYTOR-specific probes. (C) MS identification of NCL and Sam68. (JPG 1204 kb)

Additional file 8: **Figure S7.** Expression and biological function of NCL and Sam68 in CRC. (A) Higher expression of NCL in colorectal cancer than paired matched normal tissue samples from the GSE31737, GSE32323 and GSE41328 databases. (B) Higher expression of Sam68 in colorectal cancer than paired matched normal tissue samples from the GSE32323 database. (C) Decrease of the proliferation ability for NCL knockdown (siNCL) and Sam68 knockdown (siSam68) RKO cells compared with control (siNC) by CCK8. (D) Decrease of migration/invasive potentials for NCL knockdown (siNCL) and Sam68 knockdown (siSam68) RKO cells compared with control (siNC) by Transwell assay. (JPG 2659 kb)

Additional file 9: **Figure S8.** Correlation analysis of NCL, Sam68 and EMT markers in GEO GSE38832 database. (A) Correlation between NCL and EMT markers. (B) Correlation between Sam68 and EMT markers. (JPG 432 kb)

## Abbreviations

CCAL: colorectal cancer-associated lncRNA
CCAT1: colon cancer associated transcript 1
ChIRP-MS: Chromatin Isolation by RNA Purification-mass spectrometry
co-IP: Co-Immunoprecipitation
CRC: colorectal cancer
CYTOR: cytoskeleton regulator RNA/Linc00152
DFS: disease-free survival
EGFR: epidermal growth factor receptor
EMT: Epithelial-mesenchymal transition
EZH2: enhancer of zeste 2 polycomb repressive complex 2 subunit
FISH: RNA fluorescence in situ hybridization
FL: Full length
FN1: fibronectin 1
GEO: gene expression omnibus
HOTAIR: HOX transcript antisense RNA
IHC: immunohistochemistry
lncRNAs: Long non-coding RNAs
MMP9: matrix metallopeptidase 9
NCL: Nucleolin
OS: overall survival
qRT-PCR: Quantitative-Reverse transcription polymerase chain reaction-Reverse transcription polymerase chain reaction
RBDs: RNA binding domains
RBPs: RNA-binding proteins
RFS: recurrence-free survival
RIP: RNA immunoprecipitation
RT-PCR: Reverse transcription polymerase chain reaction
Sam68: KH RNA binding domain containing, signal transduction associated 1/KHDRBS1
TCGA: The Cancer Genome Atlas
WT: wild-type
ΔC: C-terminus-deleted
ΔEXON1: EXON1 deletion
ΔEXON4: EXON4 deletion
ΔKH: KH-domain-deleted
ΔN: N-terminus-deleted
