# Supplementary material for: The long non-coding RNA CYTOR drives colorectal cancer progression by interacting with NCL and Sam68
Source: Mol Cancer. 2018 Jul 31;17:110. doi: 10.1186/s12943-018-0860-7 (PMC6069835; doi:10.1186/s12943-018-0860-7)
Supplement: Supplementary file 1 — Supplementary Materials and Methods. (DOCX 33 kb) [file 12943_2018_860_MOESM1_ESM.docx]

**Supplementary Materials and Methods**

**Cell Culture and Cell Lines**

Human CRC cell lines HCT116, SW480, SW620 and RKO were purchased from American Type Culture Collection (ATCC, Manassas, VA, USA) and cultured in RPMI 1640 supplemented with 10% fetal bovine serum (Invitrogen, USA). HCT8 and HEK293T was purchased from Chinese Academy of Sciences cell bank (Shanghai, China) and cultured in RPMI 1640 and DMEM supplemented with 10% fetal bovine serum and grown at 37 °C in an atmosphere of 95% air and 5% CO2.

**Oligos and vectors**

All oligos, siRNA, shRNA and primers are included in the Table S1. The full-length CDS or various deletion mutants of CYTOR, NCL and Sam68 were cloned from human cDNA using RT-PCR and were

then ligated into pcDNA3.1, pcDNA3.1-FLAG and pcDNA3.1-HA. The Sam68-△KH, CYTOR-△exon1 and CYTOR-△exon4 were constructed by Genscript (Nanjing, China). All of the vectors were confirmed by DNA sequencing. And the transfection were conducted using Lipofectamine 2000 (Invitrogen) according to the manufacturer's protocol. All experiments were performed after 48 h of transfection.

**Cell proliferation assay**

CCK8 and soft agar colony assays were performed to investigate cell proliferation. CCK8 is briefly described that 2500 cells were seeded in 96-well plates and incubated with 100µl [medium](javascript:void(0);). An aliquot of 10 µl of CCK8 (Boster, Wuhan, China) was added to the wells and incubated for 3 h. The absorbance at 450 nm was measured to calculate the numbers of viable cells in each well. The colony assay was briefly that , an underlay containing an equal mixture of 1.0% agar and 2× medium plus 10% FBS was kept at 40°C until it was used. Two milliliters of the mixture was aliquoted into the wells of 6-well tissue culture plates in triplicate. The cells were resuspended in a mixture of equal parts of 0.8% agarose (type VII, Sigma) and 2× medium plus 10% FBS (kept at 37°C) at a final cell concentration of 5×10^4^/mL. Two milliliters of the cell suspension was aliquoted into each well. All assays were performed in triplicate.

**Transwell migration/invasion assays**

Cell motility and invasiveness were measured on Transwell and Matrigel chamber plates respectively (24-well format; 8-μm pore size; Corning Costar, New York, USA) as described previously. Images of cells on the lower face of the filter were captured in fields at 10× magnification. To quantify the cells that had migrated across the filters, we used 33% acetic acid to dissolve the crystal violet absorbed by cells on the lower face of the filter. Three independent experiments were performed in triplicate.

**RT-PCR**
Total RNAs from patient tissues or cultured cells were isolated using the Trizol (Invitrogen, USA). Then RT-PCR was performed using Takara (Dalian, China) reagent (RR047A) according to the manufacturer’s manual.

**qPCR**

Quantitative PCR (qPCR) for quantifying the expression of genes were performed using SYBR Premix Ex Taq(RR420A, TaKaRa) and cDNAs. The data were analyzed using the ΔΔCT method, which were first normalized to GAPDH. The primers used for genomic qPCR are listed.

**RNA-binding protein pull-down assay**

The RNA-binding protein pull-down assay was as in ChIRP experiments. All probes are included in the [Table S](http://www.cell.com/cms/attachment/2081749851/2072569792/mmc2.xlsx)2. Proteins was subjected to SDS page electrophoresis with silver-stained to identify by mass spectroscopy (MS) and confirmed by western blot.

**RNA immunoprecipitation**

RIP was processed according to the protocol of the Magna RIP Kit (Catalog No. 17-700, Merck Millipore, Germany) except that the beads within the Kit was replaced by M2 magnetic beads (Sigma Aldrich, St. Louis, USA) to pull down the proteins with Flag tag. Cell cultured in 15 cm plate was washed twice with 10 mL ice-cold PBS and scraped off in 1 mL PBS, collected by centrifugation at 1500 rpm for 5 min at 4°C. The cell pellet was re-suspended in an equal pellet volume of complete RIP Lysis Buffer.50 µl of M2 magnetic beads was extracted and wash twice with RIP Wash Buffer. 100 µl Lysis and beads were incubated in 900 µl RIP Immunoprecipitation Buffer overnight at 4°C. Beads were collected by magnetic separator and washed by RIP Wash Buffer by five times with shaking. The immunoprecipitate was re-suspended in 150 µl proteinase K buffer and incubated at 55°C for 30 min with shaking. After the incubation, transfer the supernatant into 250 µl RIP Wash Buffer and 400 µl of phenol:isoamyl alcohol to each tubes. After centrifugation, the aqueous phase was removed into a new tube, mixed with 400 µl chloroform. The aqueous was removed into a new tube. Salt solution was added to enhance the precipitation of RNA at -80°C for overnight. After centrifugation, the pellet was washed by 80% ethanol and re-suspended in 15 µl of RNase-free water.

**RNA fluorescence in situ hybridization**

RNA fluorescence in situ hybridization (FISH) assay was performed to identify the location of CYTOR. Fixed with 4% formaldehyde at room temperature for 10 min, added 70% ethanol and permeabilize at 2-8 °C for at least an hour. Washed with 10% formamide in 2x SSC three times and then added probe (labeled with CY5 in 5’end, oligos were list in Table S1) in hybridization buffer (10% dextran sulfate, 10% formamide in 2x SSC), incubated in a dark humidified chamber at 37 °C overnight. Finally samples were incubated in 4′,6-diamidino-2-phenylindole (DAPI, 1:1000) for 5 min counterstain. Images were obtained with Nikon confocal laser-scanning microscope.

**Immunohistochemistry**

After deparaffinization and rehydration, paraffin-embedded tissue sections (4 μm thick) were treated with retrieval buffer (pH 6.0 citrate buffer) under high pressure in a pressure cooker for 2 min. Endogenous peroxidase activity was blocked by 3% hydrogen peroxide in absolute methanol at room temperature for 15 min. Serum blocking was performed using 10% normal goat serum (Lianke Biology Technology Co., Hangzhou, China) for 30 min. Then the sections were incubated with primary antibody overnight at 4°C followed by incubation with secondary antibodies (Polink-2 Plus IHC Detection System, GBI, Bothell, WA, USA) following the manufacturer’s instructions. Staining was visualized with diaminobenzidine (ZhongshanGoldenbridge Biotechnology, Beijing, China). Then, sections were counterstained with hematoxylin, dehydrated, and then covered with coverslip.

**Western blot**

The protein expression was measured by western blot. Antibodies against NCL (K1513, Santa Crus, Dallsa, USA, 1:1000; 10556, proteintech, Chicago, USA, 1:1000), sam68(C1915, Santa Crus, Dallsa, USA, 1:1000;10222, proteintech, Chicago, USA, 1:1000), NF-κBp65(4764, CST, Danvers, USA, 1:1000), Phospho-NF-κB p65(3033, CST, Danvers, USA, 1:1000), HA-Tag(66006, proteintech, Chicago, USA, 1:1000; 3724, CST, Danvers, USA, 1:1000), E-cadherin (sc-71008, Santa Cruz, Dallas, USA, 1:1000), vimentin (5741S, CST, Danvers, USA, 1:1000),Flag (sc-807, Santa Cruz, Dallas, USA, 1:1000; F1804, Sigma Aldrich, St. Louis, USA, 1:2000), and GAPDH(Multi Sciences (Lianke) Biotech Co., Ltd, China, 1:5000) were used.IR Dye 680RD (926-68071, LI-COR, USA, 1:5000)and IR Dye 800CW (926-32210, LI-COR, USA, 1:5000) were used as the secondary antibodies.

**Subcutaneous tumor growth in nude mice**

For subcutaneous injections, 1×10^6^ cells were resuspendedwith100μl of 1×PBS and injected into each flank of BALB/nude mice (male, 5 weeks of age). Tumor growth was measured at an interval of 4 days after injection by using a caliper, and tumor volume was calculated according to the following formula: length×width^2^×0.5. Harvested tumor tissues were placed in liquid nitrogen and then frozen at −80°C or fixed in 10% buffered formalin, embedded in paraffin, sectioned and stained.

**Mouse tail-vein assay**

RKO cells were first transfected with pGKV5 luciferase vector and then treated with G418 at 4 μg/ml to obtain a cell line stably expressing luciferase. The stable cell line was then treated with control or CYTOR-shRNA to generate two groups for severe combined immunodeficient mice (NOD–SCID–gamma, male, 5 weeks old) tail-vein injection. Each mouse was injected with 2×10^6^cells with 100 μl PBS. Mice were detected every week for metastatic foci by bioluminescence imaging. Mice were first injected with luciferin (300 mg/kg, 5 min prior to imaging), anesthetized with 3% isoflurane, and then imaged in an IVIS spectrum imaging system (Caliper, Newton, USA). Images were analyzed with Living Image software (Caliper, Newton, USA). Bioluminescent flux (photons/s/sr/cm2) was determined for the tumors.

**Cripsr/Cas9 and donor vector**

Crispr-gRNA and pUC57 donor vector with homology arms (sequence below) were purchased from Genscript (Nanjing, China), which were co-tranfected into RKO Cells. The editing efficiency was identified using IDT Surveyor Mutation Detection Kits (Integrated DNA Technologies). Then the positive single clone cells were selected by puromycin and verified by DNA sequencing.

**>EXON1 (1001-1191)-Donor**

gaagagatgatgattttagatcttgtgaattgttatgtcagaggaatacatgcagaagaaaacaggcaccgaaatagttattaccagagcaaagtaaatctgaacacctaaaagaataagaaaatatcttatgcttctctctactctgaactgccttgaaaagagcaagaatttcatacatgagcctgtagcacattacaaaatctgagtatgtatttgaaattcaaatgcacacaaagttcatgcattttattttccagttgttctcaagactcctaaataaagcatttcccctgtgtgggcgacccctgggtcccatgtggggtcagtgtttgggtctctctgagaggatcaggaaggctgctcttccaggaagggctgcagggaccctgggaaccggaccagtccagctcacaagtatgaagagaatgtcgggagaggaagtggtggctatgagtcagcatgagtcatctcgttccaatgagaatgaaggctgaggtgtgagtatgtttctgtcatttttagggttgcttctgcagtttgccttggagtctgggctgtgggaaaggctgctgcgtcctggtgaggacactgcgtttctttcagactttgaagatctgtcgttttagaaaagtggctgcctctgtcaccagccccatggttactgtcctttagtgtgactgtcaggaggtgtttctcagtccttcgttgtaagaatgtagatgccggttgcaccttctgttgtcttggaagagactgcagtgcttggctggaaaataagctgctcgggactcctctgagaagccaaagtgaagctcagagatggaagtgggtatacttgtgctaacccagggttgctgaggttgggtgagcttccgcttctccgaggtggaggagaggcagctcctgagccatttctggcctcgtggtcagagctgcccaatttcagtgtgagaaataccagagaggcagaactttggctgccttctctaaaagcata

**>EXON4 (66753-67064)-Donor**

Cctcattgggctggaattccacagggatctctcattgggtctctttgggcctttggagaggggagtgcagggcaccacctgggcgaacacctgtgccatcctgtaagtccttgtgtgactcttcatccaagtcaacaggggcatgtaggggaagagcctcagggcccatcaggagtcagaccctgaccaggaagtcagaactgaatcaacttttcaataaacacacaggctggccccaaacaaagcagcaggcttttgggaacatctgccttttcagtctcttctgaattctgtttatgtggacaccccatgtcccgcaccaccattgccgccagcaatgtttgttgcttgccaatatttgaatagatgctgataaatttgattctctggcttattgtggcattcatgatatgtctttcccttttcagtttccatgtactagagcgactgtcagtgtggttggcagtgacctgtgtttttcatgcatgtttttgtttcagttgtttgcttattagctatctttaaacgctgtttgaaaggatatcgaatagtgtttgagggaaccctggcacgtccacacagctcatctgacatgtttaaatgtgtgttgggcttgactctctcataactcagccacgcttgccttgctgcttctcatacctctccagattgctgtagagtcagtgtgggcagactgacatttgagagaaacgtcgggcttcatctggaagcctctttccacacccaccctgatcgtggcctgtaaactgtggccccaagatctccattaaccttgggctttccactagagacatctgccatctctgctgcctccctcccctcacacatcctttactgcttcacttattcatcccataaggatttcttaggggcctgctgtgtgctggttctggagacaaaacagtggatgggaccaactcagccccggtccccacccagcacctctagccaagtctgagtgggctctgctgaagaagtg

**Supplement table S1 Oligos used in this study**

| **Oligos** | **Sequence** |
| --- | --- |
| NCL-RBD1(1-381)-F  NCL-RBD(1-381)-R  NCL-RBD(382-710)-F  NCL-RBD(382-710)-R  NCL-RBD(1-465)-F  NCL-RBD(1-465)-R  NCL-RBD(466-710)-F  NCL-RBD(466-710)-R  NCL-(1-710)-F  NCL-(1-710)-R | tgctggaattctgcagatatcATGGTGAAGCTCGCGAAGG  ccctctagatgcatgctcgagCTATGGTTTCTCTAGTTTAA TTTCATTGC  tgctggaattctgcagatatcATGAAAGGAAAAGACAGTAAGAAAGAGC  ccctctagatgcatgctcgagCTATTCAAACTTCGTCTTCTTTCCTTG  tgctggaattctgcagatatcATGGTGAAGCTCGCGAAGG  ccctctagatgcatgctcgagCTATCCAGTATAGTACAGGGAAATAGATCG  tgctggaattctgcagatatcATGGAGAAAGGTCAAAATCAAGACT  ccctctagatgcatgctcgagCTATTCAAACTTCGTCTTCTTTCCTTG  tgctggaattctgcagatatcATGGTGAAGCTCGCGAAGG  ccctctagatgcatgctcgagCTATTCAAACTTCGTCTTCTTTCCTTG |
| SAM68(1-443)-F  SAM68(1-443)-R  SAM68(1-346)-F  SAM68(1-346)-R  SAM68(103-443)-F  SAM68(103-443)-R | accgagctcggatccgaattcATGCAGCGCCGGGACGAC  gtagggccctctagactcgagATAACGTCCATATGGGTGCTCTC  accgagctcggatccgaattcATGTACCTGCCCGAACTCATGG  gtagggccctctagactcgagTTAATAACGTCCATATGGGTGCTC  accgagctcggatccgaattcATGCAGCGCCGGGACGAC  gtagggccctctagactcgagCTATCTTGGTGCTGGAGCACCC |
| CYTOR-m1-F  CYTOR-m1-R  CYTOR-m2-F  CYTOR-m2-R  CYTOR-m3-F  CYTOR-m3-R  CYTOR-m4-F  CYTOR-m4-R  GAPDH-F  GAPDH-R | CGGTGCCTGATTTTTTGCCTGTCTT  AAGACAGGCAAAAAATCAGGCACCG  TCTACCTGTTTTTTTCCGATCACAG  CTGTGATCGGAAAAAAACAGGTAGA  ATGGAGGGAACCCCCTGACTGGATG  CATCCAGTCAGGGGGTTCCCTCCAT  CTTGTCCTTCCCCCCCAGCAAAACTA  TAGTTTTGCTGGGGGGGAAGGACAAG  ACCACAGTCCATGCCATCAC  TCCACCACCCTGTTGCTGTA |
| CYTOR-ΔExon1-F  CYTOR-ΔExon1-R  CYTOR-ΔExon4-F  CYTOR-ΔExon4-R | CCCAAAGTTACGGAGGACCC  AATGGGAAACCGACCAGACC  TGACATTCCAGACAAGCGGT  GAGATGGTTGCTGGGTCCTC |
| CYTOR-shRNA-S  CYTOR- shRNA –A  Scamble-S  Scramble-A | CCGGCAGTCTCTATGTGTCTTAACTCGAGTTAAGACACATAGAGACTG TTTTTTG  AATTCAAAAACAGTCTCTATGTGTCTTAACTCGAGTTAAGACACATAGAGACTG  CACCTTCTCCGAACGTGTCACGTTTCAAGAGAACGTGACACGTTCGGAGAATTTTTTG  GATCCAAAAAATTCTCCGAACGTGTCACGTTCTCTTGAAACGTGACACGTTCGGAGAA |
| EXON1-gRNA1  EXON1-gRNA2  EXON1-gRNA3  EXON4-gRNA1  EXON4-gRNA2  EXON4-gRNA3 | GGGCTCAGGCACCGCTTGTC  TTAGTCGTGTGTACATCATT  ATCTTCACAGCACAGTTCCT  GAGGCCTCTGCATTTGCGGG  ATTTTGGTCATGGGCTGGTC  TGTTGCCCGCCGATCACAGC |
| NCL-siRNA-F  NCL-siRNA-R  Sam68-siRNA-F  Sam68-siRNA-R  NC-SiRNA-F  NC-SiRNA-R  CYTOR-siRNA1-F  CYTOR-siRNA1-R  CYTOR-siRNA2-F  CYTOR-siRNA2-R | GGAAGAGCCUGUCAAAGAATT  UUCUUUGACAGGCUCUUCCTT  CGGCAGAAAUUGAGAAGAUTT  AUCUUCUCAAUUUCUGCCGTT  UUCUCCGAACGUGUCACGUTT  ACGUGACACGUUCGGAGAATT  CAGUCUCUAUGUGUCUUAATT  UUAAGACACAUAGAGACUGTT  CACACUUGAUCGAAUAUGATT  UCAUAUUCGAUCAAGUGUGTT |
| cy5-CYTOR-1 | TCCGTAACTTTGGGCATGAG |
| cy5-CYTOR-2 | AGCCATCAAAAGAAGGCCAG |
| cy5-CYTOR-3 | CAGTTATTCGAGGGATGCAG |

**Supplement table S2 The sequence of ChIRP probes**

| **Probe #** | **Probe (5'-> 3')** | **Probe Position*** | **Percent GC** |
| --- | --- | --- | --- |
| 1 | cccaatgatgtacacacgac | 29 | 50.0% |
| 2 | agatctgaagacaggcacgg | 127 | 55.0% |
| 3 | tccgtaactttgggcatgag | 302 | 50.0% |
| 4 | agccatcaaaagaaggccag | 397 | 50.0% |
| 5 | cagttattcgagggatgcag | 490 | 50.0% |
| 6 | aacaggtagaggtgctggag | 582 | 55.0% |
| 7 | caaatgcagaggcctcagag | 671 | 55.0% |
| 8 | aagacacatagagactggcc | 751 | 50.0% |

*Position of first nucleotide of probe corresponding to target sequence
