# Supplementary figures and images for: The long non-coding RNA CYTOR drives colorectal cancer progression by interacting with NCL and Sam68
Source: Mol Cancer. 2018 Jul 31;17:110. doi: 10.1186/s12943-018-0860-7 (PMC6069835; doi:10.1186/s12943-018-0860-7)

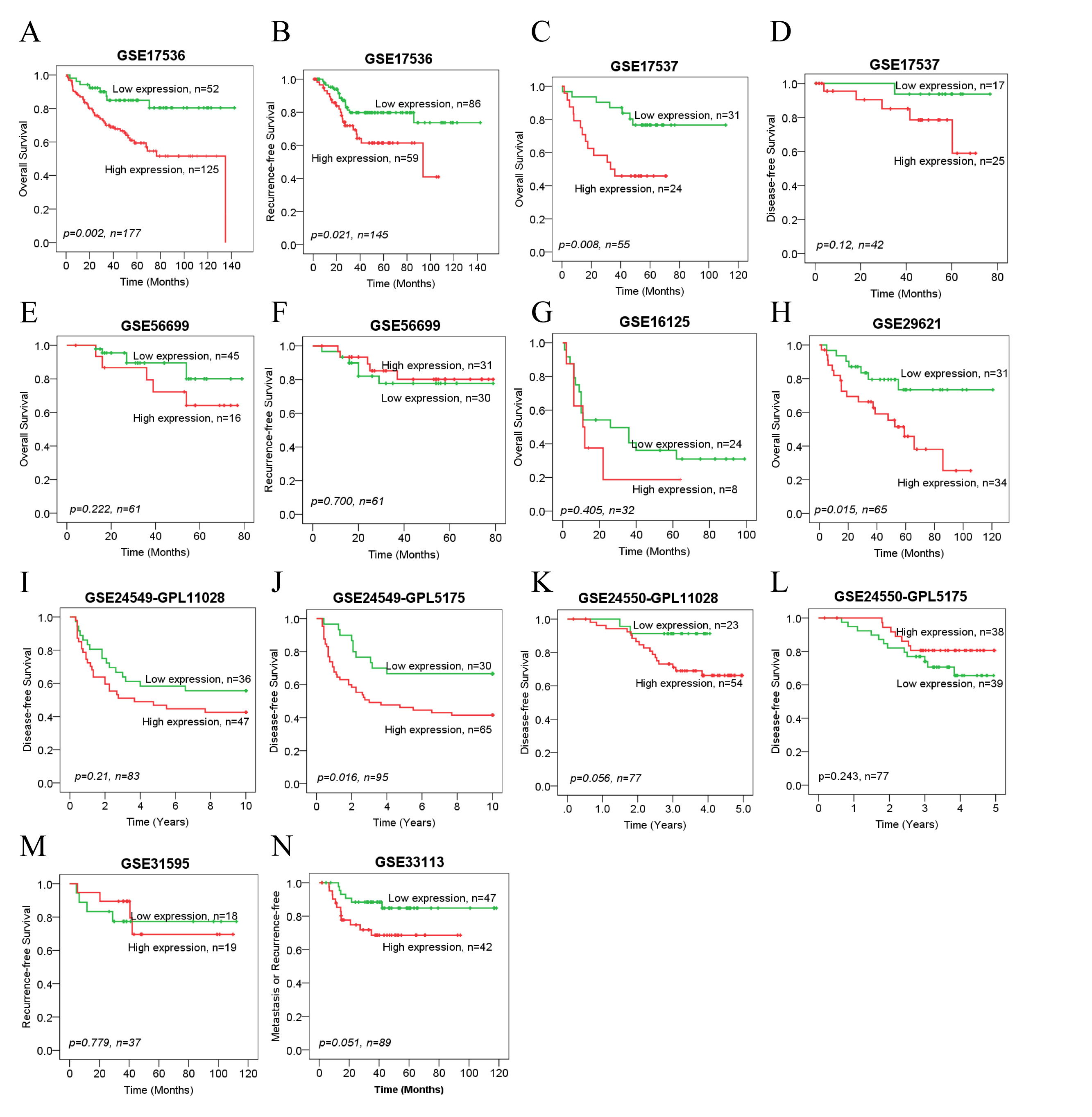

Supplement: Supplementary file 2 — Figure S1. CYTOR expression and CRC prognosis. (A, B) Kaplan-Meier plots of overall survival (A) and recurrence-free survival (B) for CRC samples from the GSE17536 database. (C, D) Kaplan-Meier plots of overall survival (C) and recurrence-free survival (D) for CRC samples from the GSE17537 database. (E, F) Kaplan-Meier plots of overall survival (E) and recurrence-free survival (F) for CRC samples from the GSE56699 database. (G, H) Kaplan-Meier plots of overall survival for CRC samples from the GSE16125 (G) and GSE29621 (H) databases. (I, J, K, L) Kaplan-Meier plots of disease-free survival for CRC samples from the GSE24549-GPL11028 (I), GSE24549-GPL5175 (J), GSE24550-GPL11028 (K) and GSE24550-GPL5175 (L) databases. (M, N) Kaplan-Meier plots of recurrence-free survival for CRC samples from the GSE31595 (M) and GSE33113 (N) databases. (JPG 688 kb) [file 12943_2018_860_MOESM2_ESM.jpg]

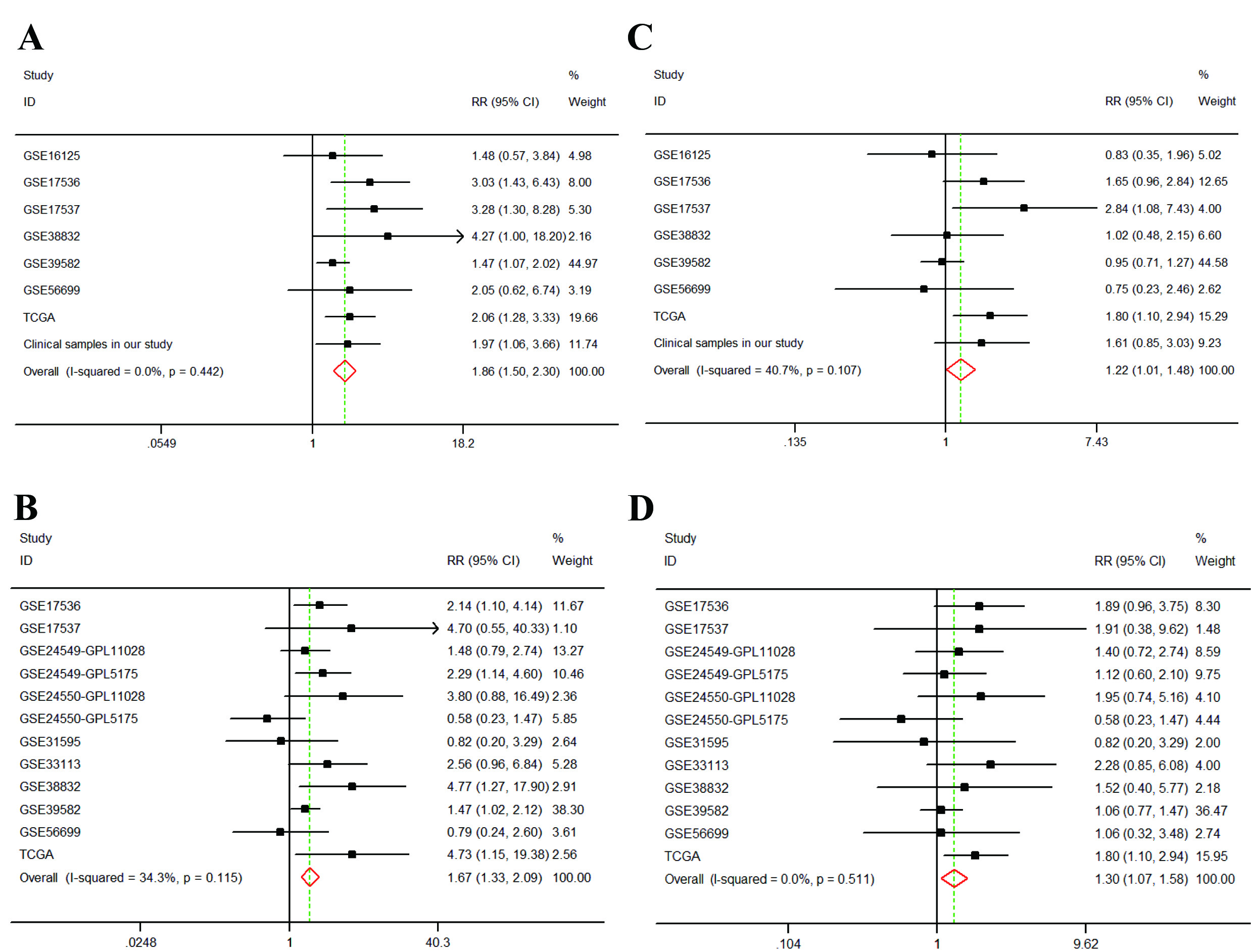

Supplement: Supplementary file 3 — Figure S2. A meta-analysis of the association between CYTOR and CRC survival. (A) Forest plots of the association between CYTOR expression and overall survival at the cutoff value set according to the ROC. (B) Forest plots of the association between CYTOR expression and disease- or recurrence-free survival at the cutoff value set according to the ROC. (C) Forest plots of the association between CYTOR expression and overall survival at the P50 cutoff value. (D) Forest plots of the association between CYTOR expression and disease- or recurrence-free survival at the P50 cutoff value. (JPG 1781 kb) [file 12943_2018_860_MOESM3_ESM.jpg]

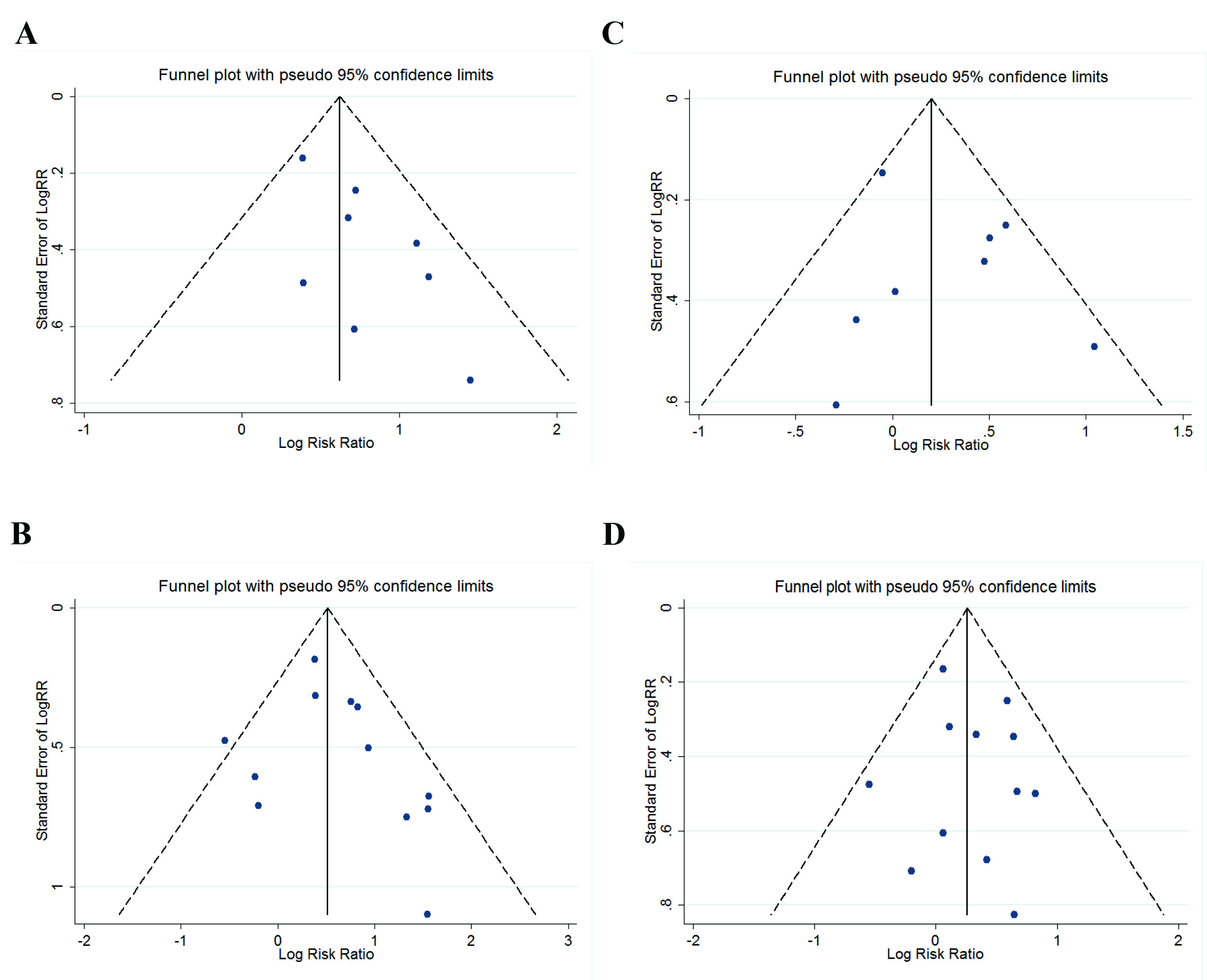

Supplement: Supplementary file 4 — Figure S3. Funnel plots for the relationship between CYTOR and CRC prognosis. (A) Funnel plots of the association between CYTOR expression and overall survival at the cutoff value set according to the ROC. (B) Funnel plots of the association between CYTOR expression and disease- or recurrence-free survival at the cutoff value set by according to the ROC. (C) Funnel plots of the association between CYTOR expression and overall survival at the P50 cutoff value. (D) Funnel plots of the association between CYTOR expression and disease- or recurrence-free survival at the P50 cutoff value. (JPG 1257 kb) [file 12943_2018_860_MOESM4_ESM.jpg]

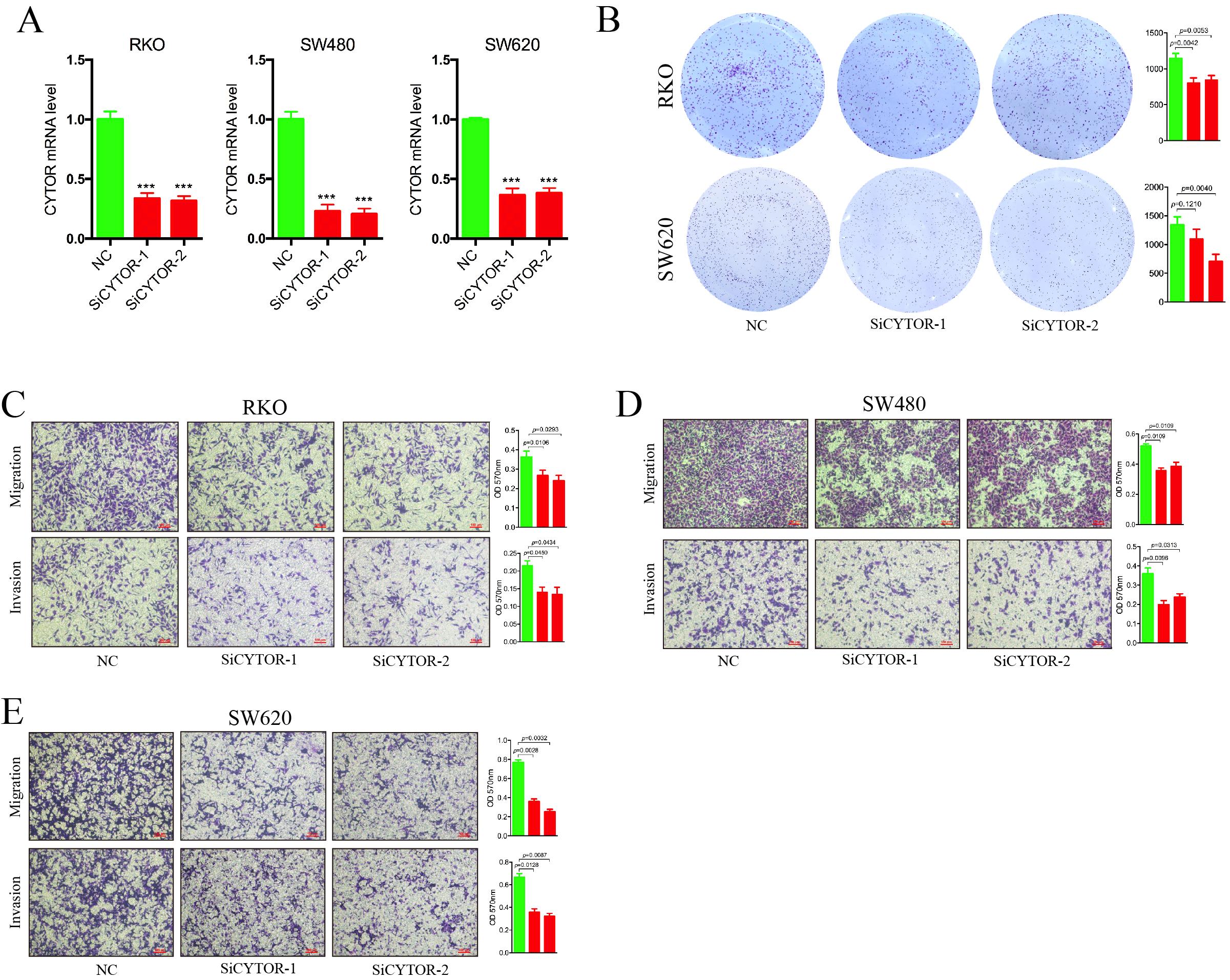

Supplement: Supplementary file 5 — Figure S4. Knockdown of CYTOR inhibited anchorage-independent growth and migration/invasion.(A) qRT-PCR for detection of CYTOR in RKO, SW480 and SW620 cells knocked known by siRNAs of CYTOR. (B) Reduction of colony formation ability for CYTOR knockdown RKO and SW620 cells by siRNAs compared with control (NC). (C, D and E) Decrease of migration/invasive potential for CYTOR knockdown RKO (C), SW480 (D) and SW620 (E) cells by siRNAs compared with control by transwell assay. (JPG 2970 kb) [file 12943_2018_860_MOESM5_ESM.jpg]

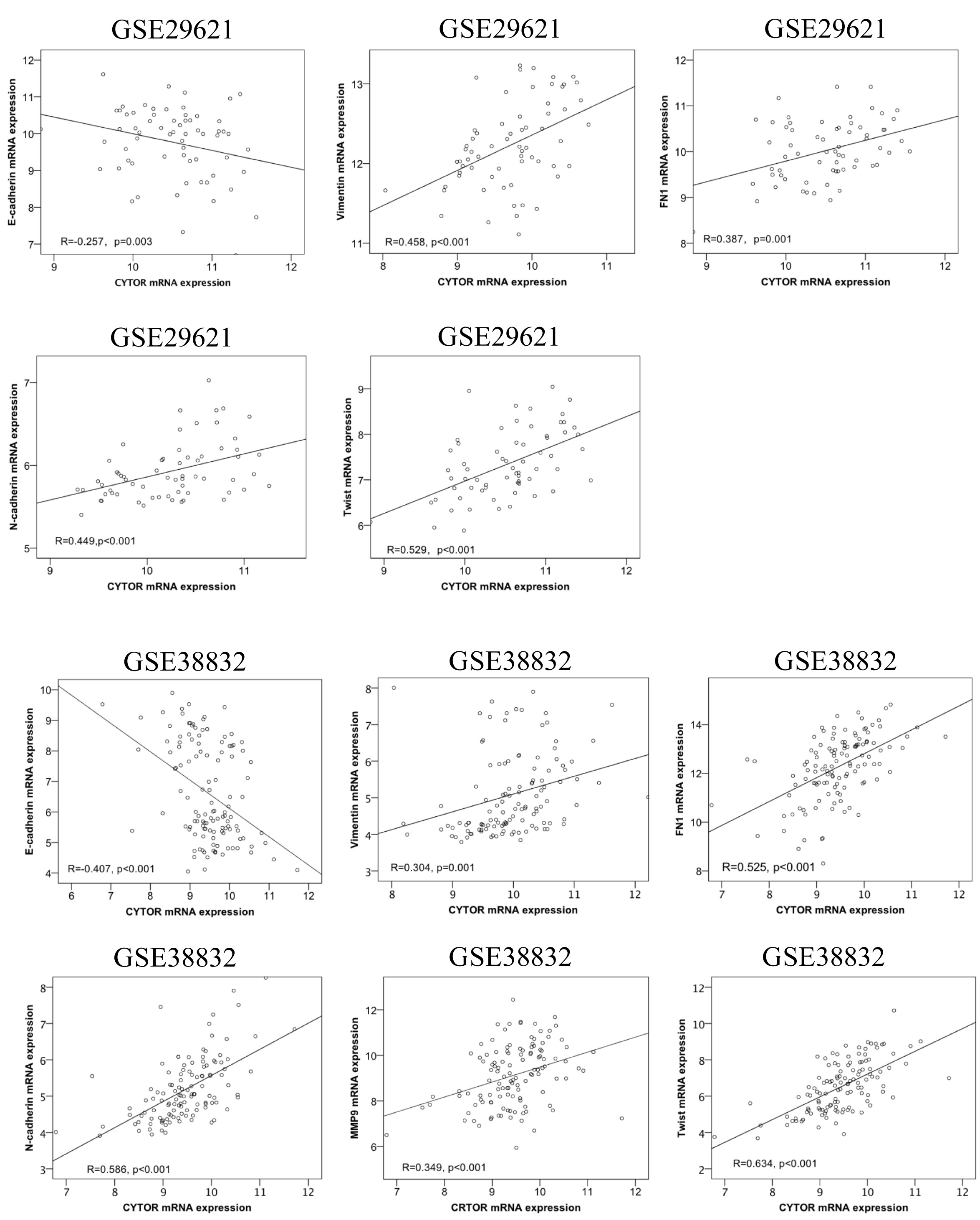

Supplement: Supplementary file 6 — Figure S5. Correlation analysis between CYTOR and EMT markers. (JPG 567 kb) [file 12943_2018_860_MOESM6_ESM.jpg]

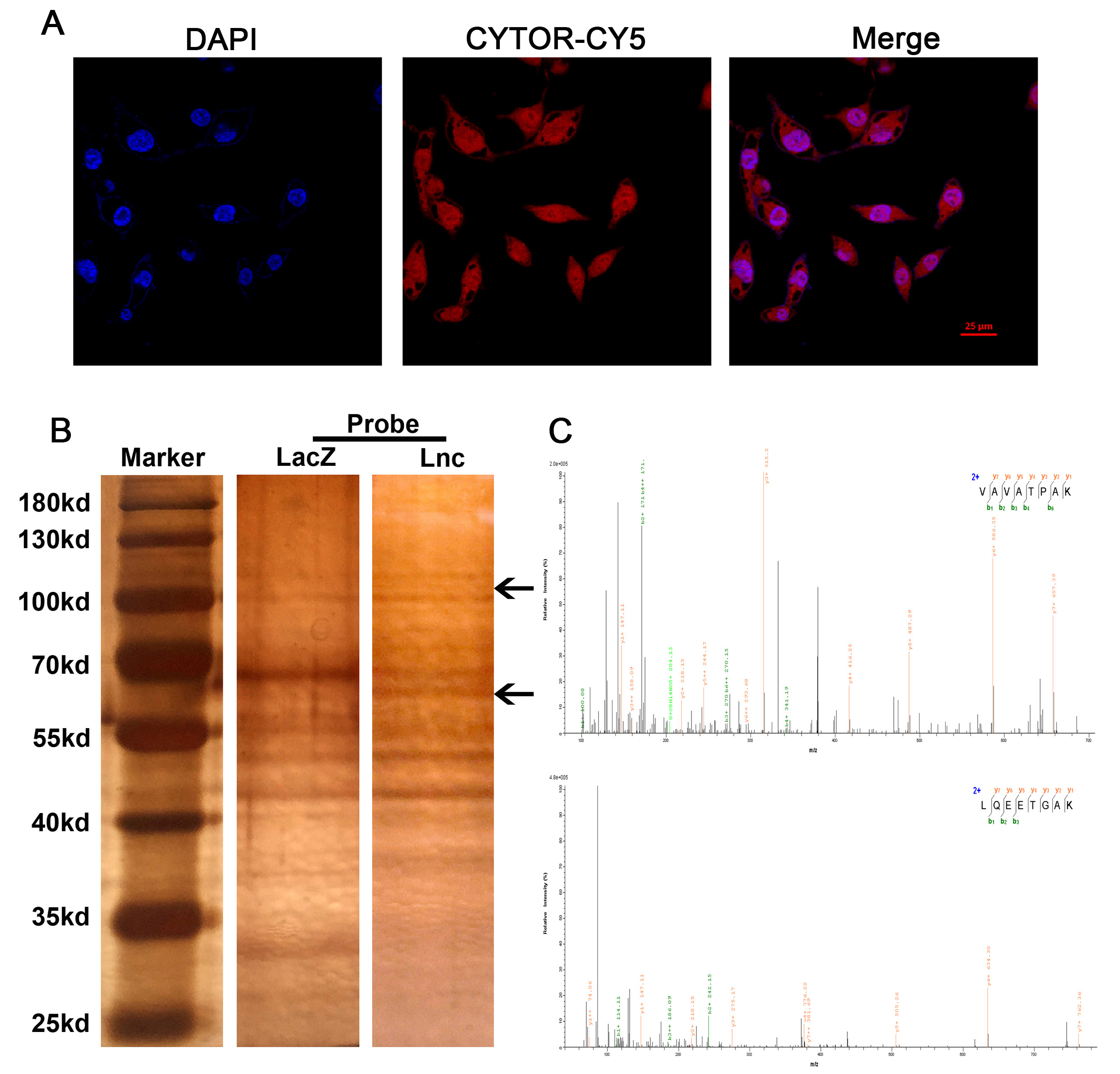

Supplement: Supplementary file 7 — Figure S6. CYTOR location in cells and its binding proteins identified by ChIRP and MS. (A) RNA FISH to detection CYTOR location in RKO cells. (B) SDS-PAGE for protein isolation by ChIRP with CYTOR-specific probes. (C) MS identification of NCL and Sam68. (JPG 1204 kb) [file 12943_2018_860_MOESM7_ESM.jpg]

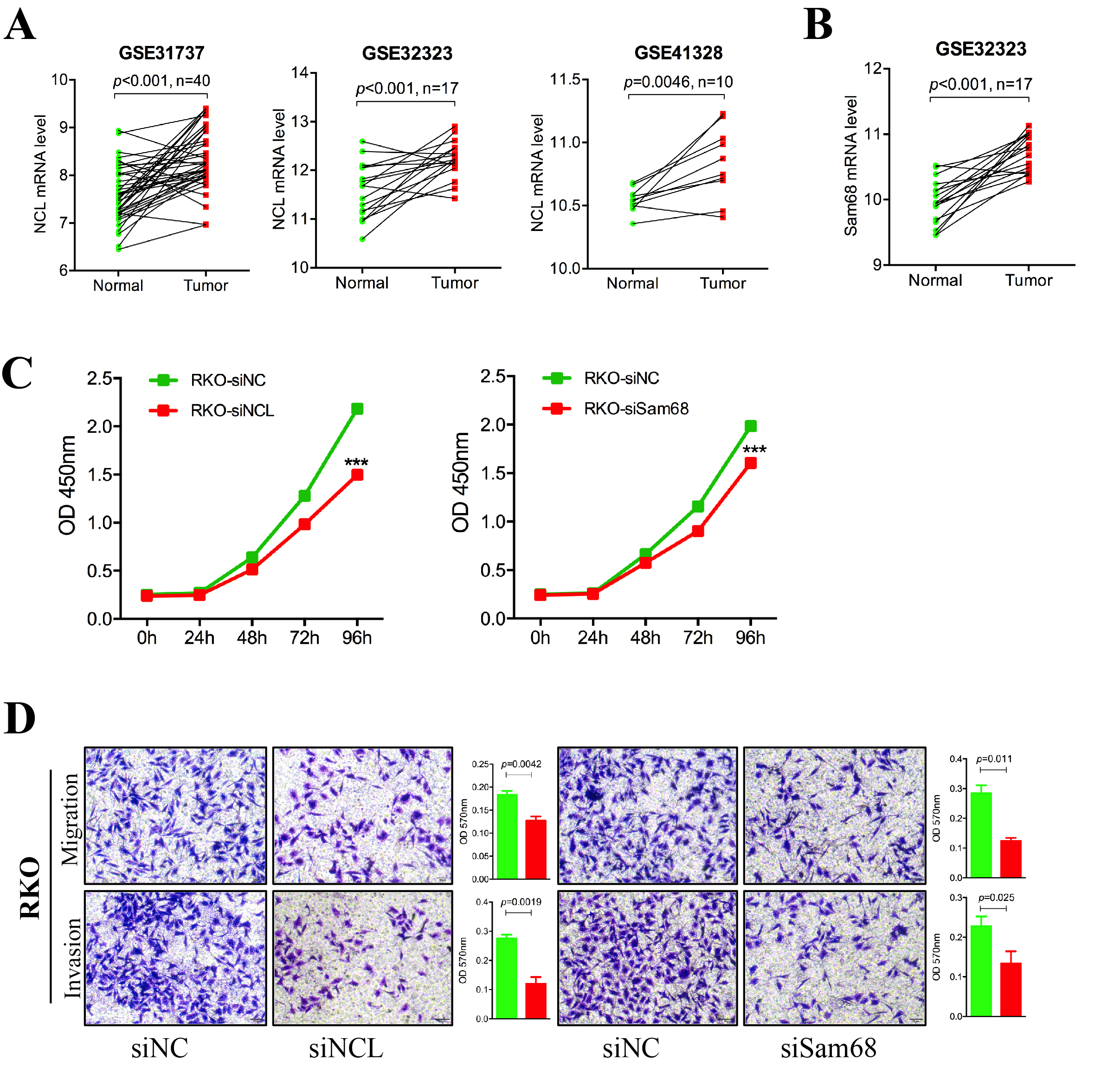

Supplement: Supplementary file 8 — Figure S7. Expression and biological function of NCL and Sam68 in CRC. (A) Higher expression of NCL in colorectal cancer than paired matched normal tissue samples from the GSE31737, GSE32323 and GSE41328 databases. (B) Higher expression of Sam68 in colorectal cancer than paired matched normal tissue samples from the GSE32323 database. (C) Decrease of the proliferation ability for NCL knockdown (siNCL) and Sam68 knockdown (siSam68) RKO cells compared with control (siNC) by CCK8. (D) Decrease of migration/invasive potentials for NCL knockdown (siNCL) and Sam68 knockdown (siSam68) RKO cells compared with control (siNC) by Transwell assay. (JPG 2659 kb) [file 12943_2018_860_MOESM8_ESM.jpg]

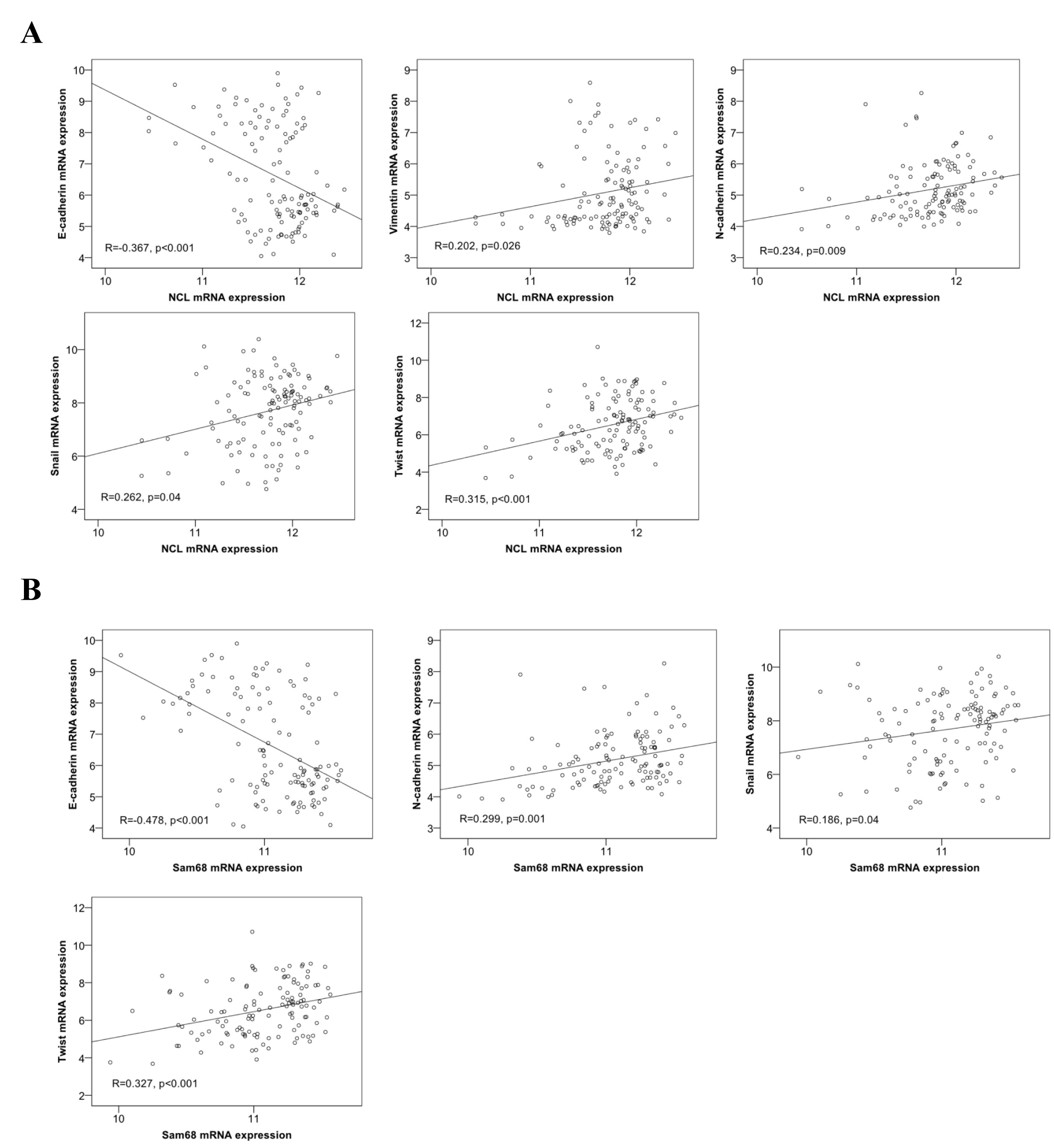

Supplement: Supplementary file 9 — Figure S8. Correlation analysis of NCL, Sam68 and EMT markers in GEO GSE38832 database. (A) Correlation between NCL and EMT markers. (B) Correlation between Sam68 and EMT markers. (JPG 432 kb) [file 12943_2018_860_MOESM9_ESM.jpg]
